# Supplementary material for: Early-to-mid stage idiopathic Parkinson’s disease shows enhanced cytotoxicity and differentiation in CD8 T-cells in females
Source: Nat Commun. 2023 Nov 20;14:7461. doi: 10.1038/s41467-023-43053-0 (PMC10662447; doi:10.1038/s41467-023-43053-0)
Supplement: Supplementary file 1 — Supplementary Information [file 41467_2023_43053_MOESM1_ESM.pdf]

Early-to-mid stage idiopathic Parkinson's disease shows enhanced cytotoxicity and differentiation in CD8 T-cells in females

Supplementary Information

Christophe M. Capelle[1, 2, §], Séverine Ciré[1, £], Fanny Hedin[3], Maxime Hansen[4, 5], Lukas Pavelka[4, 5, 6], Kamil Grzyb[4], Dimitrios Kyriakis[4,¥], Oliver Hunewald[1], Maria Konstantinou[3], Dominique Revets[3], Vera Tslaf[1, 2, 6], Tainá M. Marques[6], Clarissa P. C. Gomes[4], Alexandre Baron[1], Olivia Domingues[1], Mario Gomez[3], Ni Zeng[1,2], Fay Betsou[7,8], Patrick May[4], Alexander Skupin[4,9,10], Antonio Cosma[3], Rudi Balling[4,11], Rejko Krüger[4, 5, 6], Markus Ollert[1, 12,*], Feng Q. Hefeng[1,13, *]

[1] Department of Infection and Immunity, Luxembourg Institute of Health (LIH), 29, rue Henri Koch, L-4354, Esch-sur-Alzette, Luxembourg
[2] Faculty of Science, Technology and Medicine, University of Luxembourg, 2, avenue de Université, L-4365, Esch-sur-Alzette, Luxembourg
[3] National Cytometry Platform, Luxembourg Institute of Health, 29, rue Henri Koch, L-4354 Esch-sur-Alzette, Luxembourg
[4] Luxembourg Centre for Systems Biomedicine (LCSB), University of Luxembourg, 6, avenue du Swing, L-4367, Belvaux, Luxembourg
[5] Parkinson Research Clinic, Centre Hospitalier de Luxembourg (CHL), 4, Rue Nicolas Ernest Barblé, L-1210, Luxembourg, Luxembourg
[6] Transversal Translational Medicine, Luxembourg Institute of Health, 1A-B, rue Thomas Edison, L-1445, Strassen, Luxembourg
[7] Integrated Biobank of Luxembourg (IBBL), 1, rue Louis Rech, L-3555, Dudelange, Luxembourg
[8] CRBIP, Institut Pasteur, Université Paris Cité, Paris, France
[9] Department of Physics and Material Science, 162a, av. de la Faïencerie, L-1511, Luxembourg, Luxembourg
[10] Department of Neurosciences, University California San Diego School of Medicine, 9500 Gilman Drive, La Jolla, CA 92093-0662, USA
[11] Institute of Molecular Psychiatry, University of Bonn, Venusberg-Campus 1, D-53127, Bonn, Germany
[12] Department of Dermatology and Allergy Center, Odense Research Center for Anaphylaxis (ORCA), University of Southern Denmark, Odense, 5000 C, Denmark
[13] Data Integration and Analysis Unit, Luxembourg Institute of Health (LIH), L-1445 Strassen, Luxembourg
[§] Current address, Institute of Microbiology, ETH Zurich, Vladimir-Prelog-Weg 4, CH-8049 Zurich, Switzerland
[£] Current address, Eligo Bioscience, 111, avenue de France, 75013 Paris, France
[¥] Current address, Icahn School of Medicine at Mount Sinai, New York, NY 10029, USA

* Corresponding author. Correspondence to M.O. (Markus.ollert@lih.lu) or F.Q.H. (feng.he@lih.lu)

Supplementary Note 1: Extended acknowledgement to the consortium members of the cohort used in this work.

We would like to thank all the anonymous participants of the Luxembourg Parkinson's Study for their important support of our research. Furthermore, we acknowledge the joint effort of the National Centre of Excellence in Research on Parkinson's Disease (NCER-PD) Consortium members from the partner institutions Luxembourg Centre for Systems Biomedicine, Luxembourg Institute of Health, Centre Hospitalier de Luxembourg, and Laboratoire National de Santé generally contributing to the Luxembourg Parkinson's Study as listed below: Geeta ACHARYA[2], Gloria AGUAYO[2], Myriam ALEXANDRE[2], Muhammad ALI[1], Wim AMMERLANN[2], Giuseppe ARENA[1], Rudi BALLING[1], Michele BASSIS [1], Roxane BATUTU 3, Katy BEAUMONT[2], Regina BECKER[1], Camille BELLORA[2], Guy BERCHEM[3], Daniela BERG[11], Alexandre BISDORFF[5], Ibrahim BOUSSAAD[1], Kathrin BROCKMANN[11], Jessica CALMES[2], Lorieza CASTILLO[2], Gessica CONTESOTTO[2], Nancy DE BREMAEKER 3, Nico DIEDERICH[3], Rene DONDELINGER[5], Daniela ESTEVES [2], Guy FAGHERAZZI [2], Jean-Yves FERRAND [2], Manon GANTENBEIN [2], Thomas GASSER[11], Piotr GAWRON[1], Soumyabrata GHOSH[1], Marijus GIRAITIS[2,3], Enrico GLAAB[1], Elisa GÓMEZ DE LOPE[1], Jérôme GRAAS [2], Mariella GRAZIANO[17], Valentin GROUES[1], Anne GRÜNEWALD[1], Wei GU[1], Gaël HAMMOT[2], Anne-Marie HANFF[2], Linda HANSEN[1,3], Michael HENEKA[1], Estelle HENRY[2], Sylvia HERBRINK[6], Sascha HERZINGER[1], Michael HEYMANN[2], Michele HU[8], Alexander HUNDT[2], Nadine JACOBY[18], Jacek JAROSLAW LEBIODA[1], Yohan JAROZ[1], Sonja JÓNSDÓTTIR[2], Quentin KLOPFENSTEIN [1], Jochen KLUCKEN[1,2,3], Rejko KRÜGER[1,2,3], Pauline LAMBERT[2], Zied LANDOULSI [1], Roseline LENTZ[7], Inga LIEPELT[11], Robert LISZKA[14], Laura LONGHINO[3], Victoria LORENTZ[2], Paula Cristina LUPU[2], Clare MACKAY [10], Walter MAETZLER[15], Katrin MARCUS[13], Guilherme MARQUES[2], Tainá M. MARQUES[1], Patricia MARTINS CONDE[1], Patrick MAY[1], Deborah MCINTYRE[2], Chouaib MEDIOUNI[2], Francoise MEISCH[1], Myriam MENSTER[2], Maura MINELLI[2], Michel MITTELBRONN[1,4], Brit MOLLENHAUER[12], Friedrich MÜHLSCHLEGEL[4], Romain NATI[3], Ulf NEHRBASS[2], Sarah NICKELS[1], Beatrice NICOLAI[3], Jean-Paul NICOLAY[19], Fozia NOOR [2], Marek OSTASZEWSKI[1], Clarissa P. C. GOMES[1], Sinthuja PACHCHEK[1], Claire PAULY[1,3], Laure PAULY[1], Lukas PAVELKA[1,3], Magali PERQUIN[2], Rosalina RAMOS LIMA[2], Armin RAUSCHENBERGER [1], Rajesh RAWAL[1], Dheeraj REDDY BOBBILI[1], Kirsten ROOMP[1], Eduardo ROSALES [2], Isabel ROSETY[1], Estelle SANDT[2], Stefano SAPIENZA[1], Venkata SATAGOPAM[1], Margaux SCHMITT[2], Sabine SCHMITZ[1], Reinhard SCHNEIDER[1], Jens SCHWAMBORN[1], Amir SHARIFY[2], Ekaterina SOBOLEVA[1], Kate SOKOLOWSKA[2], Hermann THIEN[2], Elodie THIRY[3], Rebecca TING JIIN LOO[1], Christophe TREFOIS[1], Johanna TROUET[2], Olena TSURKALENKO[2], Michel VAILLANT[2], Mesele VALENTI[2], Carlos VEGA[1], Liliana VILAS BOAS[3], Maharshi VYAS[1],

Richard WADE-MARTINS[9], Paul WILMES[1], Evi WOLLSCHEID-LENGELING[1], Gelani ZELIMKHANOV[3]. The affiliations of individuals are listed as follows. [1] Luxembourg Centre for Systems Biomedicine, University of Luxembourg, Esch-sur-Alzette, Luxembourg; [2] Luxembourg Institute of Health, Strassen, Luxembourg; [3] Centre Hospitalier de Luxembourg, Strassen, Luxembourg; [4] Laboratoire National de Santé, Dudelange, Luxembourg; [5] Centre Hospitalier Emile Mayrisch, Esch-sur-Alzette, Luxembourg; [6] Centre Hospitalier du Nord, Ettelbrück, Luxembourg; [7] Parkinson Luxembourg Association, Leudelange, Luxembourg; [8] Oxford Parkinson's Disease Centre, Nuffield Department of Clinical Neurosciences, University of Oxford, Oxford, UK; [9] Oxford Parkinson's Disease Centre, Department of Physiology, Anatomy and Genetics, University of Oxford, Oxford, UK; [10] Oxford Centre for Human Brain Activity, Wellcome Centre for Integrative Neuroimaging, Department of Psychiatry, University of Oxford, Oxford, UK; [11] Center of Neurology and Hertie Institute for Clinical Brain Research, Department of Neurodegenerative Diseases, University Hospital Tübingen, Tübingen, Germany; [12] Paracelsus-Elena-Klinik, Kassel, Germany; [13] Ruhr-University of Bochum, Bochum, Germany; [14] Westpfalz-Klinikum GmbH, Kaiserslautern, Germany; [15] Department of Neurology, University Medical Center Schleswig-Holstein, Kiel, Germany; [16] Department of Neurology Philipps, University Marburg, Marburg, Germany; [17] Association of Physiotherapists in Parkinson's Disease Europe, Esch-sur-Alzette, Luxembourg; [18] Private practice, Ettelbruck, Luxembourg; [19] Private practice, Luxembourg, Luxembourg.

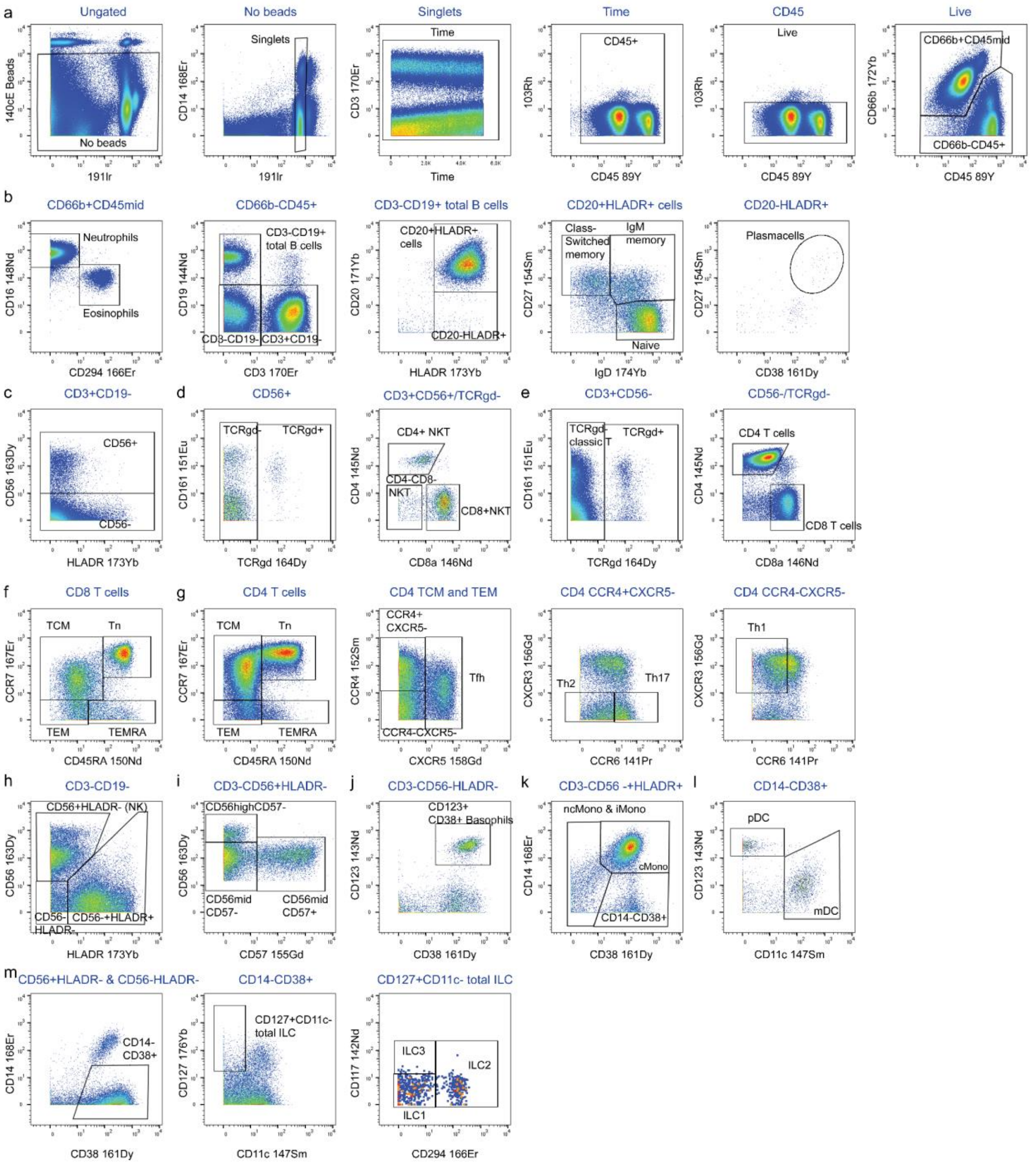

Supplementary Fig. 1. CyTOF gating strategy used in this study.

The gating strategy of the CyTOF analysis for various peripheral immune (sub-)populations from whole blood. The CyTOF markers used in this study are provided in **Supplementary Table 3**. Labels for some of the major subsets are enlarged. The corresponding parent gate was labelled in blue above the corresponding cytometry plot. To increase the explorability, we divided the overall gating strategy into different subpanels, including the initial general gating (singlet, living cells etc.) (**a**), B cell subsets and neutrophils/eosinophils gates (**b**), gates separating CD56+/CD56- cells (**c**), natural killer T (NKT) subsets (**d**), gdT cells as well as total CD4 and CD8 T cells (**e**), CD8 naïve/memory subsets (**f**), CD4 subsets (**g**), natural killer (NK)

starting gates (**h**), NK subsets (**i**), basophils (**j**), monocyte subsets (**k**), dendritic cell (DC) subsets (**l**) and innate lymphoid cell (ILC) subsets (**m**). cMono, classical monocytes; iMono, intermediate monocytes; ncMono, non-classical monocytes; mDC, myeloid DC; pDC, plasmacytoid DC; Tn, naïve T cells; TCM, central memory T cells; TEM, effector memory T cells; TEMRA, terminally-differentiated effector memory T cells; Th1/Th2/Th17, type 1/2/17 T helper cells; Tfh, T follicular helper cells.

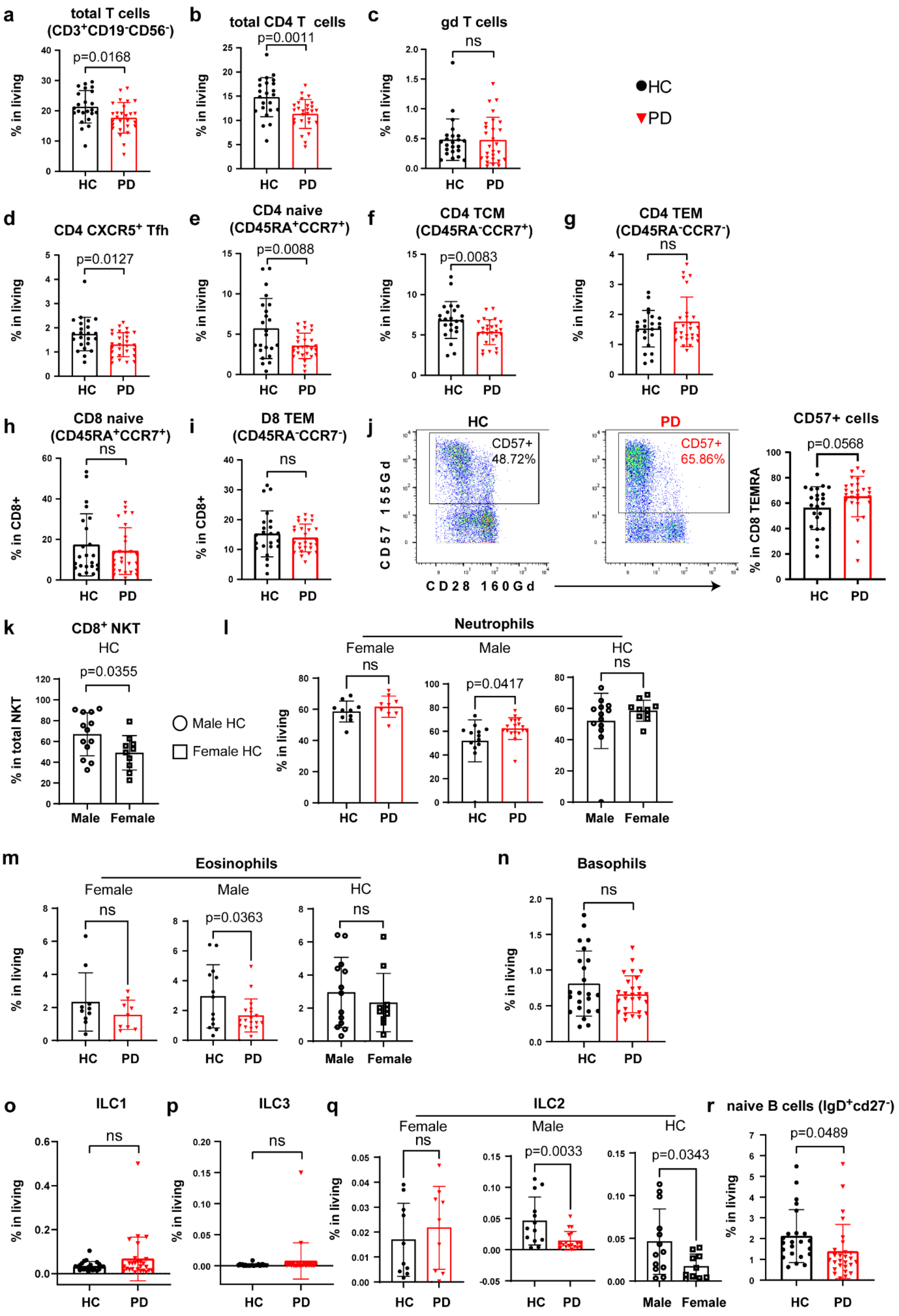
a total T cells (CD3+CD19-CD56-)
p=0.0168
b total CD4 T cells
p=0.0011
c gd T cells
ns
●HC
▼PD
d CD4 CXCR5+ Tfh
p=0.0127
e CD4 naive (CD45RA+CCR7+)
p=0.0088
f CD4 TCM (CD45RA-CCR7+)
p=0.0083
g CD4 TEM (CD45RA-CCR7-)
ns
h CD8 naive (CD45RA+CCR7+)
ns
i D8 TEM (CD45RA-CCR7-)
ns
j HC
CD57+ 48.72%
PD
CD57+ 65.86%
CD57 155Gd
CD28 160Gd
CD57+ cells
p=0.0568
% in CD8 TEMRA
k CD8+ NKT
HC
p=0.0355
% in total NKT
Male HC
Female HC
l Neutrophils
Female
ns
Male
p=0.0417
HC
ns
m Eosinophils
Female
ns
Male
p=0.0363
HC
ns
n Basophils
ns
o ILC1
ns
p ILC3
ns
q ILC2
Female
ns
Male
p=0.0033
HC
p=0.0343
r naive B cells (IgD+cd27-)
p=0.0489
% in living
% in CD8+
HC
PD
Male
Female

Supplementary Fig. 2. Extended analysis on major immune subsets examined by CyTOF.

a-c Scatter dot plots showing the frequency of total CD3 T cells ($CD3^{+}CD19^{-}CD56^{-}$) (**a**), total CD4 T cells (**b**) and γδ T cells (**c**) among living $CD45^{+}$ cells in PD and HC, as analyzed by CyTOF. **d-g** Scatter dot plots showing the frequency of circulating CD4 $CXCR5^{+}$ T follicular help (Tfh) (**d**), $CD45RA^{+}CCR7^{+}$ naïve (**e**), $CD45RA^{-}CCR7^{+}$ central memory (TCM) (**f**) and $CD45RA^{-}CCR7^{-}$ effector memory (TEM) (**g**) T cells. **h, i** Scatter dot plots showing the frequency of $CD45RA^{+}CCR7^{+}$ naïve (**h**) and $CD45RA^{-}CCR7^{-}$ effector memory (TEM) (**i**) among CD8 T cells. (**j**) Scatter dot plots showing the frequency of $CD57^{+}$ cells among CD8 TEMRA ($CD45RA^{+}CCR7^{-}$). Left panel, representative cytometry plots showing the expression of CD57 and CD28 in CD8 TEMRA. **k** Scatter dot plots showing the frequency of $CD8^{+}$ NKT among total NKT between male and female HC. **l**, **m**, **q** Scatter dot plots showing the frequency of neutrophils (**l**), eosinophils (**m**) or ILC2 (**q**) among living $CD45^{+}$ singlets in female (left) or male (middle) participants. The comparison between female and male HC was displayed in the right subpanel. **n-p, r** Scatter dot plots showing the frequency of basophils (**n**), ILC1 (**o**), ILC3 (**p**) and naïve B cells (**r**) among living $CD45^{+}$ immune cells. For detailed gating strategy, please refer to **Supplementary Fig. 1**. All the statistical description of major immune subsets was also provided in **Supplementary Table 5**. The combination of markers defining the corresponding subset was directly displayed in the panel title (**a-i**). The results were analyzed using unpaired two-tailed Student's *t* test. Data are presented as mean of the given group± standard deviation (s.d.). Each symbol represents the measurement from one individual participant (**a-r**). ns, not significant; all significant P-values are indicated. HC, healthy controls, n=24; PD, patients with Parkinson's disease, n=28. Of note, one PD and one HC were excluded due to technical failure during CyTOF staining. Female HC, n=10; female PD, n=9; male HC, n=13; male PD, n=18. NKT, Natural killer cells; ILC, innate lymphoid cells. Source data are provided as a Source Data file.

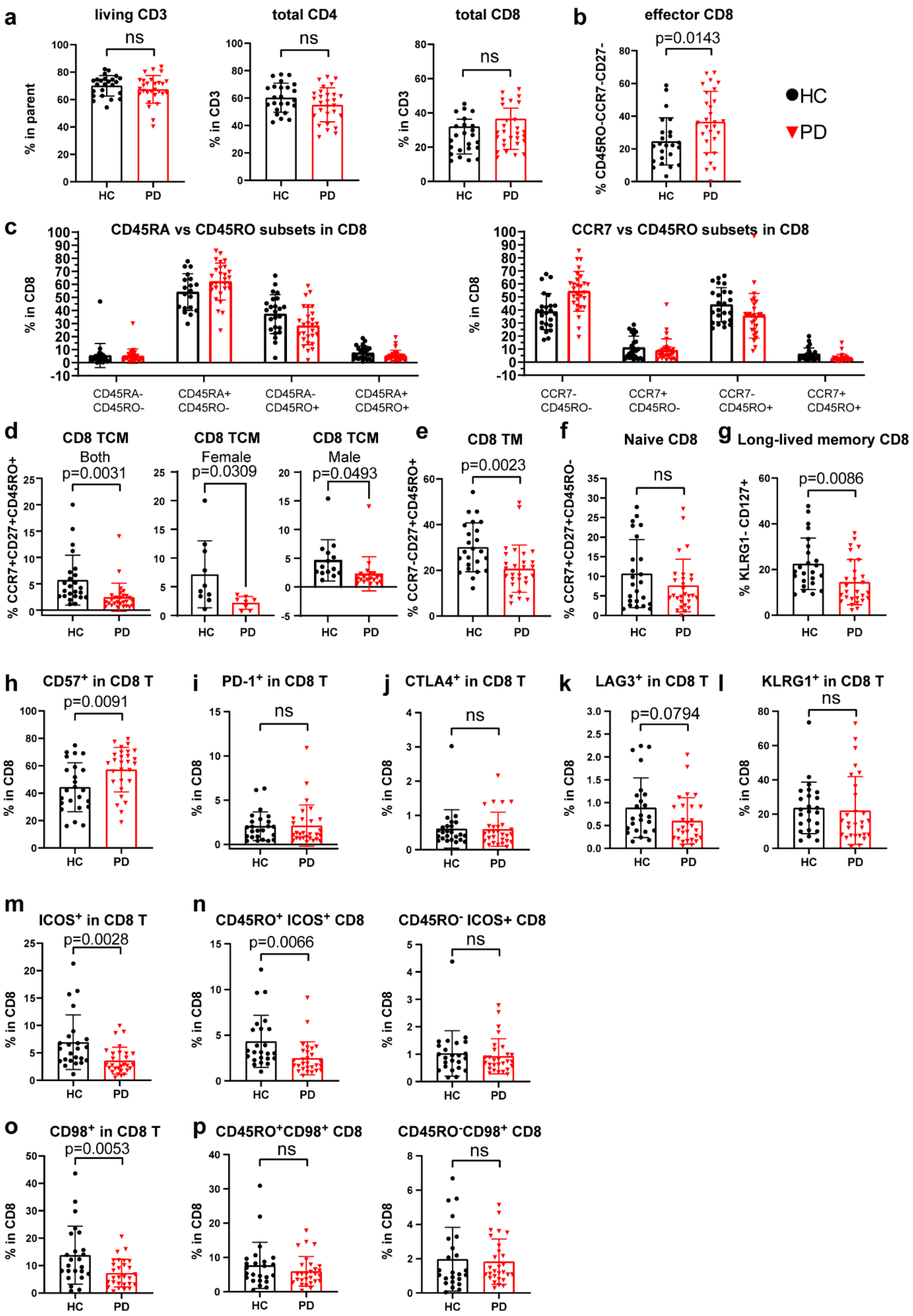
a
living CD3
ns
% in parent
total CD4
ns
% in CD3
total CD8
ns
% in CD3
b
effector CD8
p=0.0143
% CD45RO-CCR7-CD27-
HC
PD
c
CD45RA vs CD45RO subsets in CD8
% in CD8
CD45RA- CD45RO-
CD45RA+ CD45RO-
CD45RA- CD45RO+
CD45RA+ CD45RO+
CCR7 vs CD45RO subsets in CD8
% in CD8
CCR7- CD45RO-
CCR7+ CD45RO-
CCR7- CD45RO+
CCR7+ CD45RO+
d
CD8 TCM
Both
p=0.0031
% CCR7+CD27+CD45RO+
CD8 TCM
Female
p=0.0309
CD8 TCM
Male
p=0.0493
e
CD8 TM
p=0.0023
% CCR7-CD27+CD45RO+
f
Naive CD8
ns
% CCR7+CD27+CD45RO-
g
Long-lived memory CD8
p=0.0086
% KLRG1- CD127+
h
CD57+ in CD8 T
p=0.0091
% in CD8
i
PD-1+ in CD8 T
ns
% in CD8
j
CTLA4+ in CD8 T
ns
% in CD8
k
LAG3+ in CD8 T
p=0.0794
% in CD8
l
KLRG1+ in CD8 T
ns
% in CD8
m
ICOS+ in CD8 T
p=0.0028
% in CD8
n
CD45RO+ ICOS+ CD8
p=0.0066
% in CD8
CD45RO- ICOS+ CD8
ns
% in CD8
o
CD98+ in CD8 T
p=0.0053
% in CD8
p
CD45RO+CD98+ CD8
ns
% in CD8
CD45RO-CD98+ CD8
ns
% in CD8
HC
PD

Supplementary Fig. 3. Early-to-mid stage iPD shows fewer memory CD8 T cells and displays no sign of accelerated exhaustion.

a Scatter dot plots showing the frequency of total CD3, CD4 and CD8 T cells in PD and HC as analyzed by FCM. The parent gate of CD3+ cells is living lymphocyte singlets. **b** Scatter dot plots showing the frequency of effector CD8 T cells among total CD8 T cells. **c** Scatter dot plots showing the proportions of CD45RA vs CD45RO (left) and CCR7 vs CD45RO (right) quartered subpopulations of CD8 T cells. **d-g** Scatter dot plots showing the frequency of TCM (central memory) (**d**) for all (left) or females (middle) or males (right), TM (transitional memory) (**e**), naïve (**f**) and long-lived memory (**g**) among CD8 T cells in PD and HC. The combination of markers defining the corresponding subset was directly labelled in the y-axis title. **h-l** Scatter dot plots showing the frequency of $CD57^+$ (**h**), $PD\text{-}1^+$ (**i**), $CTLA4^+$ (**j**), $LAG3^+$ (**k**) and $KLRG1^+$ (**l**) populations among CD8 T cells. **m, n** Scatter dot plots showing the frequency of $ICOS^+$ cells (**m**), $ICOS^+CD45RO^+$ or $ICOS^+CD45RO^-$ cells (**n**) among total CD8 T cells in PD and HC. **o, p** Scatter dot plots showing the frequency of total $CD98^+$ populations (CD98: amino acid transporter) (**o**) as well as $CD98^+CD45RO^+$ or $CD98^+CD45RO^-$ cells (**p**) among total CD8 T cells. The results were analyzed using unpaired two-tailed Student *t* test. Data are presented as mean of the given group± standard deviation (s.d.). Each symbol represents the measurement from one individual (**a-p**). ns, not significant; all significant P-values are indicated (except for **c**, where the purpose was only to show the relative average proportions of each of the four subsets). HC, healthy controls, n=24; PD, patients with Parkinson's disease, n=28. Female HC, n=10; female PD, n=8; male HC, n=14; male PD, n=19. Of note, for **d**, one female PD sample was excluded as the same patient visited twice within a short period. Source data are provided as a Source Data file.

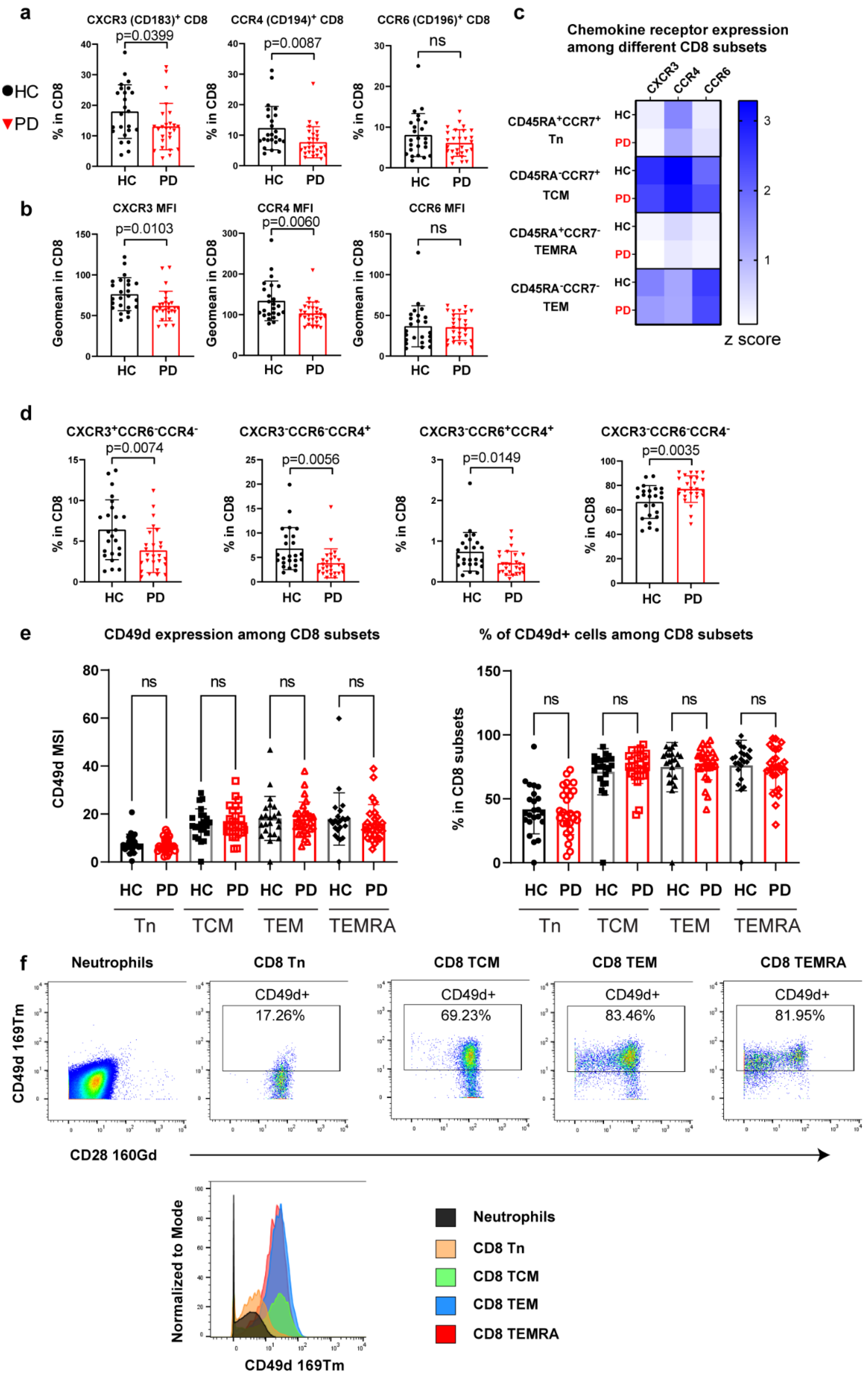

a
CXCR3 (CD183)+ CD8
p=0.0399
CCR4 (CD194)+ CD8
p=0.0087
CCR6 (CD196)+ CD8
ns
HC
PD
% in CD8
b
CXCR3 MFI
p=0.0103
CCR4 MFI
p=0.0060
CCR6 MFI
ns
Geomean in CD8
c
Chemokine receptor expression among different CD8 subsets
CXCR3
CCR4
CCR6
CD45RA+CCR7+ Tn
CD45RA-CCR7+ TCM
CD45RA+CCR7- TEMRA
CD45RA-CCR7- TEM
z score
d
CXCR3+CCR6-CCR4-
p=0.0074
CXCR3-CCR6-CCR4+
p=0.0056
CXCR3-CCR6+CCR4+
p=0.0149
CXCR3-CCR6-CCR4-
p=0.0035
e
CD49d expression among CD8 subsets
CD49d MSI
% of CD49d+ cells among CD8 subsets
% in CD8 subsets
Tn
TCM
TEM
TEMRA
f
Neutrophils
CD8 Tn
CD8 TCM
CD8 TEM
CD8 TEMRA
CD49d+
17.26%
69.23%
83.46%
81.95%
CD49d 169Tm
CD28 160Gd
Normalized to Mode

Supplementary Fig. 4. CD8 TEMRA show normal expression of analyzed chemokine receptors and CD49d in early-to-mid stage iPD.

a Scatter dot plots showing the frequency of CXCR3/CD183, CCR4/CD194 and CCR6/CD196 positive cells among total CD8 T cells. **b** Scatter dot plots showing the geometric mean (geomean, reflecting MFI) of CXCR3, CCR4 and CCR6 among total CD8 T cells. **c** Heatmap showing the averaged expression levels of the analyzed chemokine receptors in subpopulations of CD8 T cells for the given group. The frequency of cells expressing the given chemokine receptor was normalized along column. CCR7 expression was not shown because CCR7 was used to define CD8 naïve/memory T-cell subsets. **d** Scatter dot plots showing the frequency of CD8 T cells expressing different combinations of the chemokine receptors CXCR3, CCR4 and CCR6. **e** Scatter dot plots showing the expression level (MSI) of the brain homing factor CD49d (left) or the frequency of $CD49d^{+}$ cells (right) among different CD8 T-cell subsets. **f** Representative cytometry plots showing the expression of CD49d and CD28 among different cell types. Neutrophils were used as the negative controls here as they are known to be largely $CD49d^{-}$ in humans (Massena et al. 2015). Lower panel, histogram overlay of CD49d expression between different cell (sub) types. Data are presented as mean ± standard deviation (s.d.). Each symbol represents the measurement from one individual participant (**a, b, d, e**). The results were analyzed using unpaired two-tailed Student's *t* test (**a, b, d**) while **e** was analyzed using ordinary one-way ANOVA test with two-stage linear step-up procedure of Benjamini/Krieger/Yekutieli correction. q-values (FDR) were analyzed for **e**. ns, not significant; all significant p-values are indicated. HC, healthy controls, n=24; PD, patients with Parkinson's disease, n=28; FCM, flow cytometry; MSI, median signal intensity; Tn, naïve T cells; TCM, central memory; TEM, effector memory. Of note, the CD49d expression was analyzed by CyTOF while the expression of the chemokine receptors was done using FCM. For FCM, two PD samples were excluded for CD183 related results where CD183 abs was missing during staining. Source data are provided as a Source Data file.

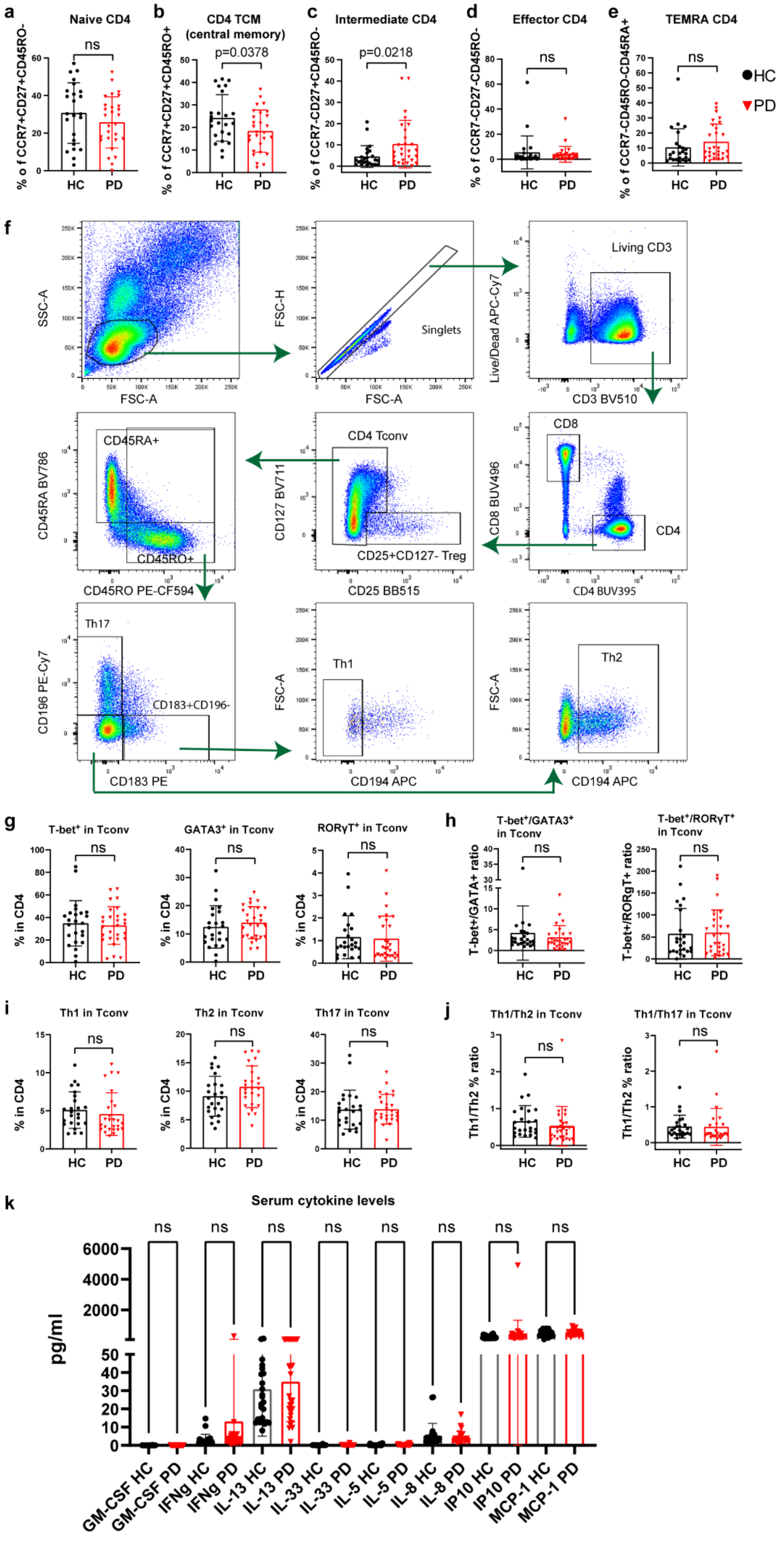
a
Naive CD4
% o f CCR7+CD27+CD45RO-
ns
HC
PD
b
CD4 TCM (central memory)
% o f CCR7+CD27+CD45RO+
p=0.0378
c
Intermediate CD4
% o f CCR7-CD27+CD45RO-
p=0.0218
d
Effector CD4
% o f CCR7-CD27-CD45RO-
e
TEMRA CD4
% o f CCR7-CD45RO-CD45RA+
HC
PD
f
SSC-A
FSC-A
FSC-H
Singlets
Living CD3
Live/Dead APC-Cy7
CD3 BV510
CD45RA+
CD45RO+
CD45RA BV786
CD45RO PE-CF594
CD4 Tconv
CD127 BV711
CD25+CD127- Treg
CD25 BB515
CD8
CD8 BUV496
CD4
CD4 BUV395
Th17
CD196 PE-Cy7
CD183+CD196-
CD183 PE
Th1
FSC-A
CD194 APC
Th2
g
T-bet+ in Tconv
GATA3+ in Tconv
RORγT+ in Tconv
% in CD4
h
T-bet+/GATA3+ in Tconv
T-bet+/GATA+ ratio
T-bet+/RORγT+ in Tconv
T-bet+/RORgT+ ratio
i
Th1 in Tconv
Th2 in Tconv
Th17 in Tconv
j
Th1/Th2 in Tconv
Th1/Th2 % ratio
Th1/Th17 in Tconv
k
Serum cytokine levels
pg/ml
GM-CSF HC
GM-CSF PD
IFNg HC
IFNg PD
IL-13 HC
IL-13 PD
IL-33 HC
IL-33 PD
IL-5 HC
IL-5 PD
IL-8 HC
IL-8 PD
IP10 HC
IP10 PD
MCP-1 HC
MCP-1 PD

Supplementary Fig. 5. CD4 TCM frequency is reduced while the balance among CD4 T-helper subsets is unaffected in early-to-mid stage iPD.

a-e Scatter dot plots showing the frequency of naïve (**a**), TCM (central memory) (**b**), intermediate (**c**), effector (**d**) and TEMRA (**e**) among CD4 T cells quantified by FCM. **f** Gating strategy to define CD4 Th1, Th2 and Th17 subsets based on the combinations of the expression of the chemokine receptors CXCR3/CD183, CCR4/CD194 and CCR6/CD196 using flow-cytometry analysis. Arrows indicate the gating workflow. **g** Scatter dot plots showing the expression of the master transcriptions factor (T-bet, GATA3 or RORγT) of CD4 T helper subset in PD and HC. **h** Ratios between T-bet$^+$/GATA3$^+$ and T-bet$^+$/RORγT$^+$ CD4 T cells in PD and HC, reflecting the ratios of Th1/Th2 and Th1/Th17, respectively. **i** Scatter dot plots showing the frequency of Th1 (CXCR3$^+$CCR6$^-$CCR4$^-$), Th2 (CXCR3$^-$CCR6$^-$CCR4$^+$) and Th17 (CXCR3$^-$CCR6$^+$) cells based on the combinations of the expression of the chemokine receptors CXCR3, CCR4 and CCR6. **j** Ratios of Th1/Th2 and Th1/Th17 cells in PD and HC. The ratios in **j** were calculated based on data of **i**. **k** Serological levels of other analysed cytokines/chemokines (except for those shown in **Fig. 2**) in PD and HC. Some values of the participants were excluded for certain cytokines because one technical replicate was detected while another replicate was below detection range or below fit curve range. The results were analysed using unpaired two-tailed Student's *t* test (**a-e, g-j**) or using Brown-Forsythe and Welch ANOVA test with Dunnette's T3 multiple comparison test (**k**). Data are presented as mean ± standard deviation (s.d.). Each symbol represents the measurement from one individual (**a-e**, **g-j, k**). ns, not significant; all significant P-values are indicated. HC, healthy controls, n=24; PD, patients with Parkinson's disease, n=28; FCM, flow cytometry. Of note (**i, j**), two PD samples were excluded for CD183-related results where CD183 abs was missing during staining. Source data are provided as a Source Data file.

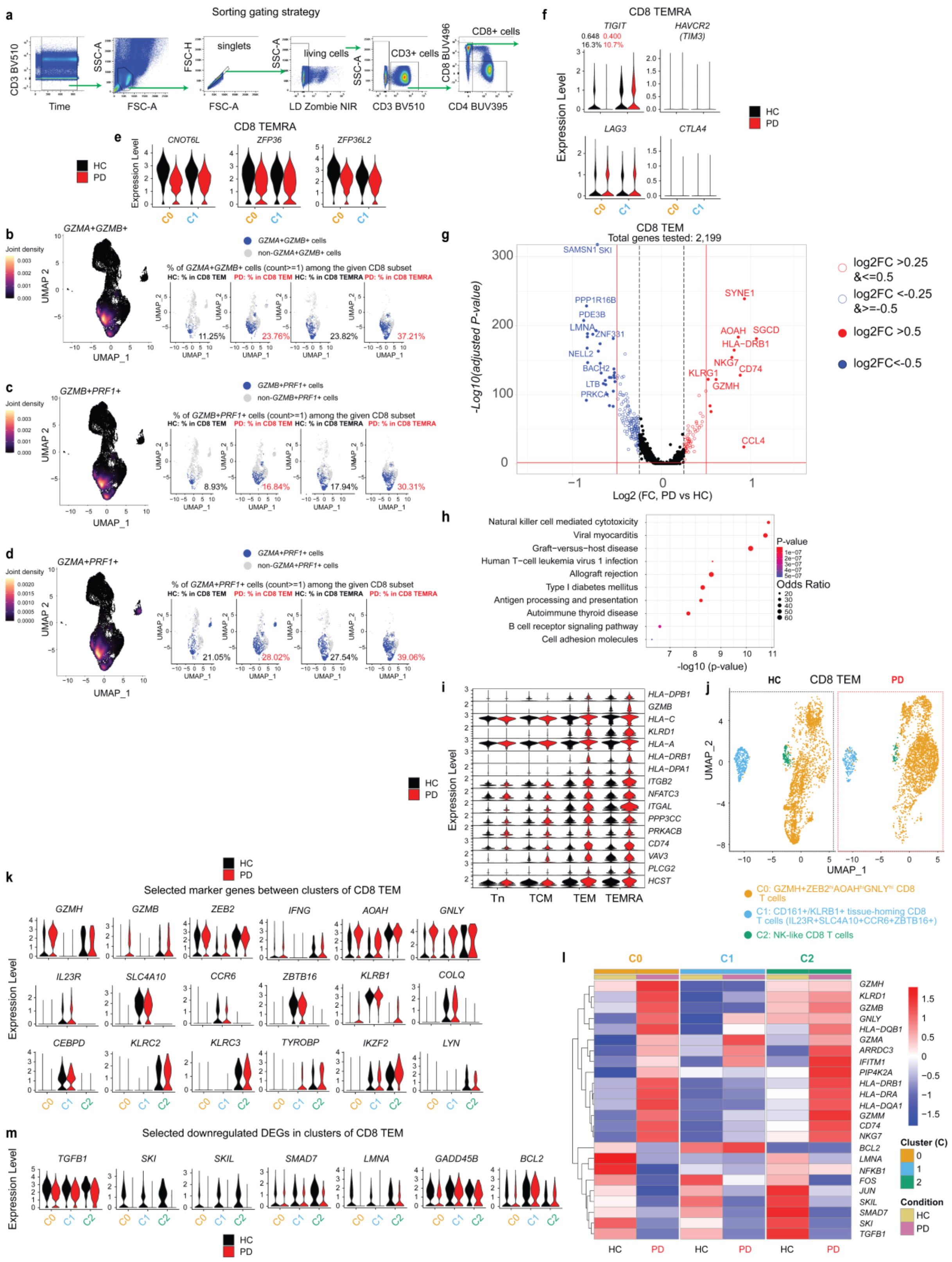

Supplementary Fig. 6. Cytotoxicity pathways are also enhanced in CD8 TEM of early-to-mid stage iPD as revealed by scRNA-seq.

a Gating strategy to sort four CD8 subsets (the last step in **Fig. 6A**). **b-d** UMAP (left) showing joint density of *GZMA* and *GZMB* (**b**), of *GZMB* and *PRF1* (**c**) and of *GZMA* and *PRF1* (**d**)

among all the CD8 T cells. Right, UMAP showing the cells co-expressing the indicated markers. For visual comparability, random downsampling was employed to display the same number of cells between different conditions and subsets. **e, f** Violin plots of selected downregulated (**e**) or exhaustion (**f**) markers in different clusters of CD8 TEMRA. The numbers under *TIGIT* indicate either the percentage of the cells expressing *TIGIT* (count>=1) or the average *TIGIT* expression in CD8 TEMRA of either PD (red) or HC (black) for Cluster 0 (C0). **g** Volcano plot showing the expression changes in CD8 TEM. The selected top up- or down-regulated genes (ranked based on gene score) were marked in red or blue labels, respectively. Vertical red solid lines indicates the log2FC value of 0.5 and -0.5, respectively, while the horizontal red line indicates –log10(0.05). **h** Top enriched KEGG pathways among upregulated genes in iPD vs HC in CD8 TEM. **i** Violin plots of selected DEGs involved in the top-enriched KEGG pathways (from **h**). **j** UMAP showing the unsupervised clustering analysis of CD8 TEM from iPD vs HC. For visual comparability, CD8 TEM cells were randomly downsampled to the same number between groups. **k, m** Violin plots of selected markers distinguishing different clusters (**k**) or selected iPD down-regulated genes (**m**) within CD8 TEM. **l** Heatmap of the selected most up- or down-regulated DEGs across different clusters in CD8 TEM of iPD vs HC. P-values in **g** and **h** were analyzed using two-tailed non-parametric Wilcoxon Rank test adjusted based on Bonferroni correction and the one-tailed Fisher's exact test, respectively. The genes selected in **k-m** were analyzed using two-tailed non-parametric Wilcoxon Rank test adjusted based on Bonferroni correction (only those with an adjusted P-value <=0.05 were considered). DEG, differentially-expressed genes; FC, fold change; UMAP, uniform manifold approximation and projection.

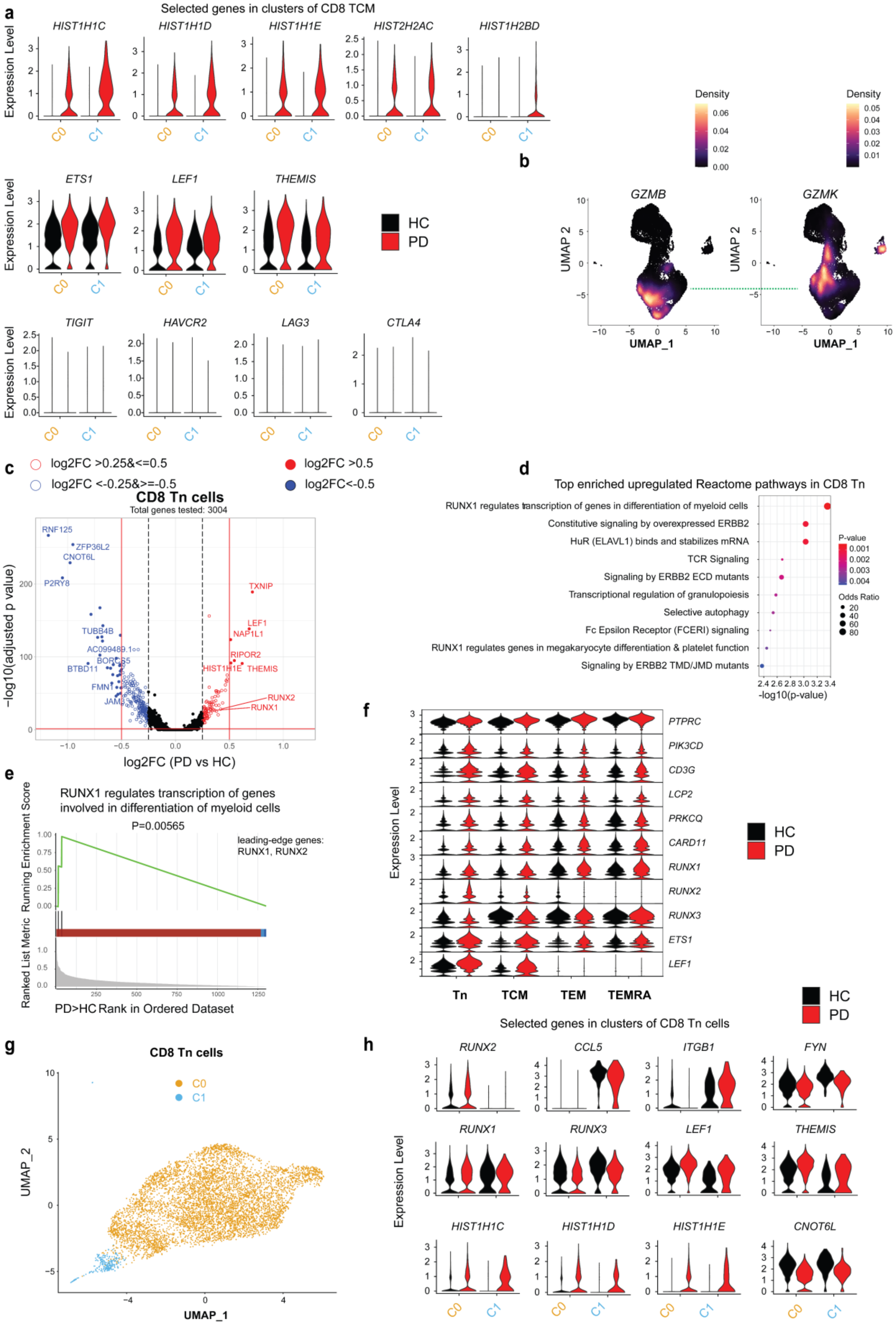
a
Selected genes in clusters of CD8 TCM
HIST1H1C
HIST1H1D
HIST1H1E
HIST2H2AC
HIST1H2BD
Expression Level
C0
C1
ETS1
LEF1
THEMIS
HC
PD
TIGIT
HAVCR2
LAG3
CTLA4
b
Density
GZMB
GZMK
UMAP 2
UMAP_1
c
log2FC >0.25&<=0.5
log2FC <-0.25&>=-0.5
log2FC >0.5
log2FC<-0.5
CD8 Tn cells
Total genes tested: 3004
RNF125
ZFP36L2
CNOT6L
P2RY8
TUBB4B
AC099489.1
BORCS5
BTBD11
FMN1
JAM3
TXNIP
LEF1
NAP1L1
RIPOR2
HIST1H1E
THEMIS
RUNX2
RUNX1
−log10(adjusted p value)
log2FC (PD vs HC)
d
Top enriched upregulated Reactome pathways in CD8 Tn
RUNX1 regulates transcription of genes in differentiation of myeloid cells
Constitutive signaling by overexpressed ERBB2
HuR (ELAVL1) binds and stabilizes mRNA
TCR Signaling
Signaling by ERBB2 ECD mutants
Transcriptional regulation of granulopoiesis
Selective autophagy
Fc Epsilon Receptor (FCERI) signaling
RUNX1 regulates genes in megakaryocyte differentiation & platelet function
Signaling by ERBB2 TMD/JMD mutants
P-value
Odds Ratio
-log10(p-value)
e
RUNX1 regulates transcription of genes
involved in differentiation of myeloid cells
P=0.00565
leading-edge genes:
RUNX1, RUNX2
Running Enrichment Score
Ranked List Metric
PD>HC Rank in Ordered Dataset
f
PTPRC
PIK3CD
CD3G
LCP2
PRKCQ
CARD11
RUNX1
RUNX2
RUNX3
ETS1
LEF1
Expression Level
Tn
TCM
TEM
TEMRA
HC
PD
g
CD8 Tn cells
C0
C1
UMAP_2
UMAP_1
h
Selected genes in clusters of CD8 Tn cells
RUNX2
CCL5
ITGB1
FYN
RUNX1
RUNX3
LEF1
THEMIS
HIST1H1C
HIST1H1D
HIST1H1E
CNOT6L
Expression Level
HC
PD

Supplementary Fig. 7. Transcriptionally-reprogramming already occurs in CD8 Tn of early-to-mid stage iPD.

a Violin plots of selected top DEGs or exhaustion marker genes in clusters of CD8 TCM. **b** UMAP showing the density of *GZMB* or *GZMK* expression in all the individual CD8 T cells analyzed by scRNA-seq. A dashed green line highlights the boundary between major *GZMB*- and *GZMK*-expressing zones. **c** Volcano plot showing the gene expression of CD8 Tn in iPD vs HC. The selected top up- or down-regulated genes (ranked based on gene score) were marked in red or blue labels, respectively. *RUNX1* and *RUNX2* were additionally labelled. Vertical red line indicates the log2FC value of 0.5 or -0.5 while the horizontal red line indicates –log10(0.05). **d** Top-ranked enriched Reactome pathways among up-regulated genes in CD8 Tn of iPD vs HC. **e** GSEA plot of the pathway showing the *RUNX1* transcription regulation involved in myeloid cell differentiation. The lower part showing the rank distribution of the genes involved in the indicated pathway. The list on the right showing the leading-edge genes. **f** Violin plots showing the genes involved in TCR signaling or early differentiation of CD8 T cells. **g** UMAP plot showing the unsupervised clustering analysis of CD8 Tn. **h** Violin plots of either selected genes distinguishing clusters or selected DEGs in iPD vs HC. P-values in **c** and **h** were analyzed using two-tailed non-parametric Wilcoxon Rank Sum test adjusted based on Bonferroni correction. In **h**, only those with an adjusted P-value <=0.05 were included. P-values in **d** and **e** were analyzed using the one-tailed Fisher's exact test and the empirical phenotype-based permutation test, respectively. Each dot represents one single cell in **b, g.** DEG, differentially-expressed genes; FC, fold change; UMAP, uniform manifold approximation and projection.

Supplementary Table 1. The exclusion criteria of the cohort.

Exclusion Criteria	
**History or presence of medication taken**	Corticosteroids<br>Cytostatic drugs<br>Immunosuppressive treatment<br>Iodine*
**Medical history**	Acute Infection<br>Autoimmune Disorders<br>Chronic Infections<br>Endocrine Diseases<br>Gastrointestinal Diseases<br>Haematological Diseases

	Immunodeficiency<br>Malignancies<br>Neurologic Diseases (other than Parkinson's disease)

* Iodine treatment could interfere with the mass cytometry (CyTOF) staining and thus was also excluded.

Supplementary Table 2. Basic demographics and clinical information of the participants in various sub-cohorts analysed in this work.

**Initial discovery cohort (fresh sampling)**			
	PD (n=28)	HC (n=24)	P-value
**Male, % (n)**	68 (19)	58 (14)	0.477 (two-tailed Chi square test)
**Age at sampling in years, mean (SD)£**	64.9 (6.97)	63.92 (3.75)	0.54 (two-tailed Student *t* test)
**Age of Onset, mean (SD)**	58.14 (9.42)	NA	NA
**Disease duration from diagnosis (years), mean (SD)**	6.64 (4.12)	NA	NA
**Disease duration from initial symptom onset, mean (SD)**	8.19 (5.37)	NA	NA
**Family History of Parkinson's Disease, % (n)**	43 (12)	NA	NA
**Hoehn and Yahr (H&Y) Staging scale, mean (SD)**	2.3 (0.42)	NA	NA
**UPDRS-III*, mean (SD)**	39.69 (13.15)	NA	NA
**LEDD¥, mean (SD)**	610.58 (344.06)	NA	NA
**MOCA€, mean (SD)**	25.21 (3.82)	NA	NA
**Validation cohort (cryopreserved PBMC; all iPD)**			
**Male, % (n)**	73 (8 out of 11)	58 (5 out of 12)	0.133 (two-tailed Chi square test)
**Age at sampling, mean (SD)**	64.90 (3.96)	63.46 (3.72)	0.38 (two-tailed Student *t* test)
**Age of Onset, mean (SD)**	59.08 (5.54)	NA	NA
**Disease duration from diagnosis (years), mean (SD)**	4.45 (3.21)	NA	NA
**Disease duration from initial symptom onset, mean (SD)**	5.82 (3.89)	NA	NA
**Hoehn and Yahr (H&Y) Staging scale, mean (SD)**	2.0 (0.27)	NA	NA
**Participants for scRNA-seq (cryopreserved PBMC; all iPD)**			
**Female,% (n)**	100 (5)	100 (4)	NA (two-tailed Chi square test)
**Age at sampling, mean (SD)**	63.14 (4.08)	62.33 (2.68)	0.77 (two-tailed Student *t* test)
**Age of Onset, mean (SD)**	54.34 (9.17)	NA	NA
**Disease duration from diagnosis (years), mean (SD)**	6.4 (7.40)	NA	NA
**Disease duration from initial symptom onset, mean (SD)**	8.8 (9.55)	NA	NA
**Hoehn and Yahr (H&Y) Staging scale, mean (SD)**	All with 2.0	NA	NA

£descriptive statistics here includes information from all idiopathic and three genetic PD.
*UPDRS-III: Motor Examination. The physician does a number of tests to rate the cardinal symptoms of PD such as rigidity, postural instability, facial expression etc.
¥LEDD: Levodopa Equivalent Daily Dose, so basically the sum of levodopa a patient is taking each day.
€MOCA: Montreal Cognitive Assessment. Provides an overall cognitive profile (0-30, with 30 meaning no cognitive deficits).
NA, no data available or not applicable; SD, standard deviation.

Supplementary Table 3. List of CyTOF antibodies (Abs) used to stain the whole blood.

Metal Isotope	Antibody	Clone	Manufacturer	Catalogue#
89Y	CD45	HI30	Fluidigm	Part of MDIPA (201325)
103Rh	Live/Dead indicator		Fluidigm	Part of MDIPA
141Pr	CD196 (CCR6)	G034E3	Fluidigm	Part of MDIPA
142Nd	CD117 (c-kit)*	104D2	Biolegend	313223
143Nd	CD123	6H6	Fluidigm	Part of MDIPA
144Nd	CD19	HIB19	Fluidigm	Part of MDIPA
145Nd	CD4	RPA-T4	Fluidigm	Part of MDIPA
146Nd	CD8a	RPA-T8	Fluidigm	Part of MDIPA
147Sm	CD11c	Bu15	Fluidigm	Part of MDIPA
148Nd	CD16	3G8	Fluidigm	Part of MDIPA
149Sm	CD45RO	UCHL1	Fluidigm	Part of MDIPA
150Nd	CD45RA	HI100	Fluidigm	Part of MDIPA
151Eu	CD161	HP-3G10	Fluidigm	Part of MDIPA
152Sm	CD194 (CCR4)	L291H4	Fluidigm	Part of MDIPA
153Eu	CD25	BC96	Fluidigm	Part of MDIPA
154Sm	CD27	O323	Fluidigm	Part of MDIPA
155Gd	CD57	HCD57	Fluidigm	Part of MDIPA
156Gd	CD183 (CXCR3)	G025H7	Fluidigm	Part of MDIPA
158Gd	CD185 (CXCR5)	J252D4	Fluidigm	Part of MDIPA
159Tb	KLRG1*	SA231A2	Biolegend	367702
160Gd	CD28	CD28.2	Fluidigm	Part of MDIPA
161Dy	CD38	HB-7	Fluidigm	Part of MDIPA
162Dy	CD336 (NKP44)*	P44-8	Biolegend	325102
163Dy	CD56 (NCAM)	NCAM16.2	Fluidigm	Part of MDIPA
164Dy	TCRγδ	B1	Fluidigm	Part of MDIPA
165Ho	CD223 (LAG3)	11C3C65	Fluidigm	3165037B
166Er	CD294	BM16	Fluidigm	Part of MDIPA
167Er	CD197 (CCR7)	G043H7	Fluidigm	Part of MDIPA

168Er	CD14	63D3	Fluidigm	Part of MDIPA
169Tm	CD49d*	9F10	Biolegend	304302
170Er	CD3	UCHT1	Fluidigm	Part of MDIPA
171Yb	CD20	2H7	Fluidigm	Part of MDIPA
172Yb	CD66b	G10F5	Fluidigm	Part of MDIPA
173Yb	HLA-DR	LN3	Fluidigm	Part of MDIPA
174Yb	IgD	IA6-2	Fluidigm	Part of MDIPA
175Lu	CD279 (PD-1)	EH12.2H7	Fluidigm	3175008B
176Yb	CD127	A019D5	Fluidigm	Part of MDIPA

***** in-house conjugation using Maxpar X8 Antibody Labeling Kits MDIPA (201325, Fluidigm). The predicted theoretical quantity (ug) used per reaction for the in-house conjugated Abs CD117-142Nd, KLRG1-159Tb, NKP44-162Dy, CD49d-169Tm, PD-1-175Lu and LAG3-165Ho was 0.5, 0.17, 0.5, 0.05, 0.17 and 0.05, respectively. The theoretical concentration was estimated based on the expected average recovery rate of 60% for an antibody conjugation procedure.

Supplementary Table 4. List of flow cytometry (FCM) Abs used to stain the PBMCs in this study.

Ab Target	Fluorochrome	Dilution	Manufacturer	Reference	Clone
**Fc Blocking Abs**	/	1:50	BD	564765	Fc1
**CD3***	BUV737	1:100	BD	741822	HIT3a
**CD3***	BV510	1:100	BD	564713	HIT3a
**CD4**	BUV395	1:100	BD	563550	SK3
**CD8**	BUV496	1:100	BD	564804	RPA-T8
**CD25**	BV786	1:50	BD	741035	2A3
**CD25**	BB515	1:50	BD	564467	2A3
**CD27**	BB700	1:50	BD	566450	M-T271
**CD28**	BUV785	1:50	BioLegend	302950	CD28.2
**CD31**	BV605	1:50	BD	562855	WM59
**CD39**	BV711	1:50	BioLegend	328228	A1
**CD45RA**	BV421	1:50	BioLegend	304130	HI100
**CD45RA**	BV785	1:50	BioLegend	304140	HI100

**CD45RO**	PE-CF594	1:50	BD	562299	UCHL1
**CD57**	FITC	1:50	BD	555619	NK-1
**CD71**	FITC	1:50	BioLegend	334104	CY1G4
**CD98**	BV786	1:50	BD	744507	UM7F8
**CD122**	PE	1:50	BioLegend	339006	TU27
**CD127 (IL7R)**	BV421	1:50	BD	562436	HIL-7R-M21
**CD127 (IL7R)**	BV711	1:50	BioLegend	351328	A019D5
**CD183 (CXCR3)**	PE	1:50	BD	560928	1C6/CXCR3
**CD194 (CCR4)**	APC	1:50	BioLegend	359408	L291H4
**CD196 (CCR6)**	PE-Cy7	1:50	BD	560620	11A9
**CD197 (CCR7)**	BV421	1:50	BioLegend	353208	G043H7
**CD223 (LAG3)**	BV711	1:50	BioLegend	369320	11C3C65
**CD278 (ICOS)**	BV605	1:50	BioLegend	313538	C398.4A
**CD279 (PD-1)**	BV605	1:50	BioLegend	329924	EH12.2H7
**GLUT1 (SLC2A1)**	PE	1:500	Abcam	ab209449	EPR3915
**KLRG1**	PE-Cy7	1:50	BioLegend	368614	14C2A07
**Intracellular markers**					
**CD152 (CTLA4)**	PE-Cy5	1:20	BD	555854	BNI3
**FOXP3**	APC	1:20	BioLegend	320114	206D
**Phospho S6**	AF488	1:20	CST	4803S	D57.2.2E
**Helios**	Pacific Blue	1:20	BioLegend	137220	22F6
**Ki-67**	Alex488	1:20	BD	561165	B56
**GATA3**	PE-Cy7	1:20	BD	560405	L50-823
**RORγT**	BV650	1:20	BD	563424	Q21-559
**T-bet**	PE	1:20	BioLegend	644810	4B10
**Eomes**	PE-Cy7	1:20	Thermo Fisher Scientific	25-4877-42	WD1928

Live/Dead	APC-Cy7	1:500	Thermo Fisher Scientific	L34976	/

*, different flurorochromes of the same markers might be used in different staining panels as we simultaneously employed five staining panels to explore.

Supplementary Table 5. CyTOF analysis reveals the percentages of major immune subsets among living CD45+ singlets or among the relevant parent gates in the peripheral fresh blood of early-to-mid stage iPD or matched HC aged 60-70 years.

No.	Items	HC (n=24), mean (SD)	PD (n=28), Mean (SD)	P-value (two-tailed t test)
	Total number of living CD45+ singlets	627629 (136842)	679999 (155232)	0.22482
	**Among living CD45+ singlets**			
**1**	ncMono plus interm Mono among living cells	0.847 (0.518)	0.75 (0.422)	0.476922
**2**	mDC among living cells	0.253 (0.065)	0.215 (0.07)	0.062098
**3**	pDC among living cells	0.111 (0.04)	0.097 (0.04)	0.24072
**4**	cMono among living cells	5.964 (1.085)	5.913 (1.37)	0.887997
**5**	Basophils among living cells	0.81 (0.445)	0.662 (0.253)	0.154289
**6**	NK among living cells	3.658 (1.6)	3.53 (1.747)	0.794596
**7**	$CD56^{high}CD57^{-}$ immature NK among living cells	0.224 (0.131)	0.16 (0.088)	0.04992
**8**	$CD56^{mid}CD57^{-}$ NK among living cells	1.619 (0.78)	1.549 (0.989)	0.788334
**9**	$CD56^{mid}CD57^{+}$ late NK among living cells	1.815 (1.174)	1.823 (1.181)	0.982319
**10**	Total ILCs among living cells	0.069 (0.042)	0.091 (0.127)	0.419163
**11**	ILC1 among living cells	0.034 (0.021)	0.067 (0.098)	0.123848
**12**	ILC2 among living cells	0.033 (0.033)	0.017 (0.015)	0.025059
**13**	ILC3 among living cells	0.001 (0.002)	0.008 (0.029)	0.290684
**14**	B cells among living cells	3.24 (1.419)	3.453 (3.736)	0.801529
**15**	$CD27^{+}CD38^{+}$ plasma cells among living cells	0.02 (0.021)	0.015 (0.01)	0.313471
**16**	$CD20^{-}HLADR^{+}$ among living cells	0.12 (0.37)	0.62 (2.09)	0.270256
**17**	$CD20^{+}HLADR^{+}$ among living cells	3.082 (1.363)	2.731 (3.217)	0.635057
**18**	$CD27^{-}IgD^{+}$ naïve B cells among living cells	2.12 (1.25)	1.379 (1.282)	0.04885
**19**	$CD27^{+}IgD^{-}$ class-switched memory B among living cells	0.395 (0.226)	0.385 (0.368)	0.911043

**20**	CD27$^+$IgD$^+$ IgM memory among living cells	0.39 (0.253)	0.781 (2.146)	0.39954
**21**	Total T cells among living cells	21.318 (5.235)	17.673 (4.946)	0.016814
**22**	TCRgd$^-$ classic T cells among living cells	20.835 (5.119)	17.198 (4.728)	0.013785
**23**	CD8+ T among living cells	5.42 (2.514)	5.372 (2.671)	0.950139
**24**	CD45RA$^-$CCR7$^-$ CD8 TEM among living cells	0.795 (0.453)	0.748 (0.43)	0.712639
**25**	CD45RA$^-$CCR7$^+$ CD8 CM among living cells	1.528 (0.963)	0.955 (0.536)	0.012486
**26**	CD45RA$^+$CCR7$^-$ CD8 TEMRA among living cells	2.165 (1.829)	2.839 (2.109)	0.247063
**27**	CD45RA$^+$CCR7$^+$ CD8 naïve T among living cells	0.783 (0.567)	0.72 (0.613)	0.713217
**28**	CD4$^+$ among living cells	14.776 (3.947)	11.321 (2.945)	0.001126
**29**	CD45RA$^-$ CD4 among living cells	8.371 (2.389)	7.099 (1.927)	0.046814
**30**	CD4 CXCR3+CCR6-CCR4-CXCR5- Th1 among living cells	1.829 (0.745)	1.636 (0.958)	0.443708
**31**	CD4 CXCR3-CCR6-CCR4+CXCR5- Th2 among living cells	1.246 (0.535)	1.01 (0.41)	0.090831
**32**	CD4 CXCR3-CCR6+CCR4+CXCR5- Th17 among living cells	0.519 (0.319)	0.549 (0.329)	0.749899
**33**	CD4 CXCR5+Tfh among living cells	1.741 (0.678)	1.304 (0.485)	0.012681
**34**	CD45RA$^-$CCR7$^-$ CD4 T among living cells	1.527 (0.594)	1.756 (0.81)	0.276958
**35**	CD45RA$^-$CCR7$^+$ CD4 T among living cells	6.848 (2.238)	5.345 (1.518)	0.008301
**36**	CD45RA$^+$CCR7$^-$ CD4 T among living cells	0.547 (0.598)	0.566 (0.647)	0.91715
**37**	CD45RA$^+$CCR7$^+$ CD4 T among living cells	5.699 (3.643)	3.55 (1.543)	0.00885
**38**	TCRgd$^+$ T among living cells	0.483 (0.341)	0.475 (0.376)	0.943847
**39**	NKT among living cells	2.407 (2.604)	2.431 (1.738)	0.969286
**40**	CD8$^+$ NKT among living cells	1.33 (1.146)	1.722 (1.345)	0.287332
**41**	CD4$^+$ NKT among living cells	0.973 (1.737)	0.546 (0.869)	0.27652
**42**	Eosinophils among living cells	2.682 (1.914)	1.628 (1.005)	0.018555
**43**	Neutrophils among living cells	54.853 (13.872)	62.153 (8.156)	0.028426
	**Among parent gate**			
**44**	ncMono plus interm Mono among CD3-CD19-CD56-+HLADR+	11.35 (5.243)	10.469 (4.895)	0.550335
**45**	cDC among CD14$^-$CD38$^+$	58.174 (5.712)	56.277 (9.996)	0.434193
**46**	pDC among CD14$^-$CD38$^+$	25.269 (6.242)	25.391 (8.386)	0.955228
**47**	cMono among CD56^{-+}HLADR$^+$	82.436 (5.429)	83.938 (5.407)	0.343294
**48**	Basophils among CD56$^-$HLADR$^-$	58.465 (18.293)	55.279 (15.374)	0.515111

**49**	NK among $CD3^{-}CD19^{-}$	28.807 (8.605)	28.745 (10.793)	0.982812
**50**	$CD56^{high}CD57^{-}$ immature NK among NK	7.04 (4.954)	6.009 (5.045)	0.47997
**51**	$CD56^{mid}CD57^{-}$ among NK	46.359 (13.894)	43.532 (15.477)	0.511909
**52**	$CD56^{mid}CD57^{+}$ late NK among NK	46.593 (16.195)	50.501 (16.552)	0.414395
**53**	Total ILCs among $CD14^{-}CD38^{+}$	1.831 (1.191)	2.776 (3.886)	0.276966
**54**	ILC1 among ILCs	56.917 (22.262)	67.578 (21.7)	0.100179
**55**	ILC2 among ILCs	40.648 (23.368)	28.852 (22.595)	0.082335
**56**	ILC3 among ILCs	2.447 (3.122)	3.641 (5.285)	0.356174
**57**	CD19 B cells among $CD66b^{-}CD45^{+}$	8.33 (3.671)	9.264 (8.883)	0.646487
**58**	$CD20^{-}HLADR^{+}$ among B cells	2.966 (7.355)	7.78 (20.574)	0.301677
**59**	$CD27^{+}CD38^{+}$ plasma cells among $CD20^{-}HLADR^{+}$ B cells	40.636 (23.249)	41.301 (22.297)	0.919915
**60**	$CD20^{+}HLADR^{+}$ among B cells	95.712 (7.352)	90.113 (22.339)	0.265394
**61**	$CD27^{-}IgD^{+}$ naïve among $CD20^{+}HLADR^{+}$	66.453 (14.11)	54.928 (20.903)	0.032729
**62**	$CD27^{+}IgD^{-}$ class-switched memory among $CD20^{+}HLADR^{+}$	13.723 (6.809)	17.912 (9.271)	0.085198
**63**	$CD27^{+}IgD^{+}$ IgM memory among $CD20^{+}HLADR^{+}$	13.893 (8.827)	17.886 (16.107)	0.303826
**64**	$CD56^{-}$ among $CD3^{+}CD19^{-}$	89.615 (7.828)	86.881 (6.587)	0.19508
**65**	$TCRgd^{-}$ classic T cells among total T cells	97.757 (1.451)	97.493 (1.734)	0.574621
**66**	$CD4^{-}CD8^{+}$ among classic T cells	25.705 (9.137)	30.118 (9.619)	0.1116
**67**	$CD45RA^{-}CCR7^{-}$ TEM among CD8	15.245 (7.516)	13.889 (4.561)	0.446601
**68**	$CD45RA^{-}CCR7^{+}$ CM among CD8	28.582 (11.591)	19.871 (10.181)	0.007914
**69**	$CD45RA^{+}CCR7^{-}$ TEMRA among CD8	36.179 (17.486)	49.607 (16.594)	0.008928
**70**	$CD45RA^{+}CCR7^{+}$ naïve among CD8	17.326 (14.998)	14.272 (11.24)	0.424869
**71**	$CD4^{+}CD8^{-}$ among classic T cells	71.145 (9.198)	66.911 (9.867)	0.132923
**72**	Th1 among CD4 $CCR4^{-}CXCR5^{-}$	61.282 (16.004)	54.486 (16.115)	0.150608
**73**	Th2 among CD4 $CCR4^{+}CXCR5^{-}$	35.101 (11.204)	34.506 (11.046)	0.854118
**74**	Th17 among CD4 $CCR4^{+}CXCR5^{-}$	14.757 (7.026)	18.987 (11.054)	0.127509
**75**	Tfh among CD4 $CD45RA^{-}$	20.455 (3.61)	18.299 (4.566)	0.079396
**76**	$CD45RA^{-}CCR7^{-}$ TEM among CD4	12.154 (8.916)	15.618 (5.296)	0.102741
**77**	$CD45RA^{-}CCR7^{+}$ CM among CD4	47.508 (12.756)	47.847 (9.05)	0.914884

**78**	CD45RA^{+}CCR7^{-} TEMRA among CD4	4.052 (4.889)	4.934 (5.083)	0.545261
**79**	CD45RA^{+}CCR7^{+} naïve among CD4	35.278 (16.482)	30.65 (10.146)	0.240098
**80**	TCRgd^{+} T among CD56^{-}	2.24 (1.452)	2.506 (1.736)	0.570601
**81**	Total NKT among CD56^{+}	85.402 (12.74)	83.294 (15.719)	0.616183
**82**	CD8^{+} NKT among NKT	59.129 (20.227)	70.188 (16.402)	0.041839
**83**	CD4^{+} NKT among NKT	33.515 (22.057)	20.707 (16.692)	0.026705
**84**	Eosinophils among CD66b^{+}CD45^{mid}	4.529 (3.466)	2.604 (1.61)	0.014893
**85**	Neutrophils among CD66b^{+}CD45^{mid}	90.811 (19.671)	97.055 (1.653)	0.114084
**86**	CD27^{+}CD38^{+} plasma cells among B cells	0.64 (0.58)	0.91 (1.11)	0.304309
**87**	CD27^{-}IgD^{+} naive among B cells	64.28 (15.04)	51.7 (23.15)	0.033506
**88**	CD27^{+}IgD^{-} class-switched among B cells	12.74 (4.82)	15.03 (8.13)	0.252162
**89**	CD27^{+}IgD^{+} IgM memory among B cells	13.01 (7.8)	15.6 (15.92)	0.489398

Supplementary Table 6. List of Abs used for the intracellular cytotoxic marker analysis via FCM and/or extracellular Abs for CD8 subset sorting via FACS.

Marker	Fluorochrome	Dilution	Manufacture	Reference	Clone	Application	Extra-cellular or intra-cellular	Lot number
CD8	BUV496	1/100	BD	612942	RPA-T8	Cytotoxic & Sorting	Extra-cellular	2213972
CD4	BUV395	1/100	BD	563550	SK3	Cytotoxic & Sorting	Extra-cellular	1313997
CD45RA	BV785	1/50	Biolegend	304140	HI100	Cytotoxic &Sorting	Extra-cellular	B369489
CD3	BV510	1/20	BD	564713	HIT3a	Cytotoxic & Sorting	Extra-cellular	2129056
CCR7	BV421	1/50	Biolegend	353208	G043H7	Cytotoxic & Sorting	Extra-cellular	B361376
CD45RO	PE-CF594	1/50	BD	562299	UCHL1	Cytotoxic& Sorting	Extra-cellular	1333677
Perforin	FITC	1/30	Biolegend	353310	B-D48	Cytotoxic	Intra-cellular	B336273
GZMB	RY586	1/750	BD	568133	GB11	Cytotoxic	Intra-cellular	2056267-1
GZMA	Alexa Fluor 700	1/150	Biolegend	507210	CB9	Cytotoxic	Intra-cellular	B322484

GZMK	Alexa Fluor 647	1/30	BD	566655	G3H69	Cytotoxic	Intra-cellular	2234680

Of note, for the extracellular and intracellular mastermix, the final volume was 50 μL and 100 uL diluted in Brilliant Stain Buffer per reaction, respectively.

Supplementary References

Massena, S., Christoffersson, G., Vågesjö, E., Seignez, C., Gustafsson, K., et al. (2015). "Identification and characterization of VEGF-A–responsive neutrophils expressing CD49d, VEGFR1, and CXCR4 in mice and humans." Blood **126**(17): 2016-2026. DOI: 10.1182/blood-2015-03-631572.
