# Supplementary material for: Early-to-mid stage idiopathic Parkinson’s disease shows enhanced cytotoxicity and differentiation in CD8 T-cells in females
Source: Nat Commun. 2023 Nov 20;14:7461. doi: 10.1038/s41467-023-43053-0 (PMC10662447; doi:10.1038/s41467-023-43053-0)
Supplement: Supplementary file 3 — Reporting Summary [file 41467_2023_43053_MOESM3_ESM.pdf]

nature portfolio

Corresponding author(s): Feng Q. Hefeng

Last updated by author(s): Oct 13, 2023

Reporting Summary

Nature Portfolio wishes to improve the reproducibility of the work that we publish. This form provides structure for consistency and transparency in reporting. For further information on Nature Portfolio policies, see our Editorial Policies and the Editorial Policy Checklist.

Statistics

For all statistical analyses, confirm that the following items are present in the figure legend, table legend, main text, or Methods section.

n/a	Confirmed	
☐	☒	The exact sample size (*n*) for each experimental group/condition, given as a discrete number and unit of measurement
☐	☒	A statement on whether measurements were taken from distinct samples or whether the same sample was measured repeatedly
☐	☒	The statistical test(s) used AND whether they are one- or two-sided<br>*Only common tests should be described solely by name; describe more complex techniques in the Methods section.*
☒	☐	A description of all covariates tested
☐	☒	A description of any assumptions or corrections, such as tests of normality and adjustment for multiple comparisons
☐	☒	A full description of the statistical parameters including central tendency (e.g. means) or other basic estimates (e.g. regression coefficient) AND variation (e.g. standard deviation) or associated estimates of uncertainty (e.g. confidence intervals)
☐	☒	For null hypothesis testing, the test statistic (e.g. *F*, *t*, *r*) with confidence intervals, effect sizes, degrees of freedom and *P* value noted<br>*Give P values as exact values whenever suitable.*
☒	☐	For Bayesian analysis, information on the choice of priors and Markov chain Monte Carlo settings
☒	☐	For hierarchical and complex designs, identification of the appropriate level for tests and full reporting of outcomes
☐	☒	Estimates of effect sizes (e.g. Cohen's *d*, Pearson's *r*), indicating how they were calculated

Our web collection on statistics for biologists contains articles on many of the points above.

Software and code

Policy information about availability of computer code

Data collection: Flow cytometry data were acquired using the BD FACSDiva™ Software (v8.0.1) and mass cytometry data were acquired using CyTOF Helios software v7.0.8493. for FACS sorting and cytotoxic analysis, data were acquired using BD FACSymphony A6 software (FACSDiva v9.5).

Data analysis: Flow cytometry data were analyzed using FlowJo V10. The supervised analysis of the CyTOF data was performed using the same FlowJo software as well. For the unsupervised analysis, the CD8 T cells were first extracted using FlowJo v10 to perform the viSNE analysis on the CellEngine (https://cellengine.com/). The viSNE analysis was achieved using all the cells from each fcs file, with 1000 iterations and a perplexity of 80. The following markers were used to generate the viSNE: CD45RA/CCR7/CD27/CD57/CD38/HLADR. The MSD cytokine/chemokine analysis was performed using the Discovery Workbench v 4.0.12 (LSR_4_0_12). All the statistical analysis including the ROC analysis, correlation analysis and visualization was done using GraphPad Prism v 9.0. For single-cell RNA-seq analysis, the sequenced libraries were aligned to the GRCh38-2020-A human reference genome using the CellRanger (V7.0.1). In droplet-based single-cell RNA-seq experiments, there is often background contamination resulting from a certain amount of background mRNAs. This contamination arises not only from the cells enclosed within a droplet, but also from the solution containing the cells, created through cell lysis. To correct this contamination, we utilized SoupX (v. 1.6.2) to estimate the cell-specific contamination fraction and adjust the expression matrix. Subsequently, we employed scDblFinder (v. 1.12.0) to identify and filter out doublets. In this process, mitochondrial (13), ribosomal (94), and hemoglobin (1) genes were removed for a further analysis. The integration of the four cell types was conducted using sctransform (v2). Density plots and joint density plots were created using Nebulosa (v1.8.0) and scCustomize (v1.1.1). Differential expression analysis was performed using the FindMarkers function from the Seurat package (v. 4.3.0). The volcano plots of the DEGs were illustrated using ggplot2 (v.3.4.1) and ggrepel (v.0.9.2). Enrichment analysis was performed on the genes showing an adjusted p-value lower than 0.05 and an average log2 fold change greater than 0.25 using the enrichR library (v.3.2). Additionally, a GSEA analysis on KEGG or Reactome pathways was conducted using clusterProfiler (v. 4.6.2). The genes were ranked based on the gene.score for the GSEA analysis. To calculate the pseudo-ordering (also known as 'pseudotime') of cells, we used slingshot (v. 2.6.0) to "adjust" a one-dimensional curve intersecting with the cell

subsets in the multi-dimensional expression space. We utilized low-dimensional PC coordinates for noise reduction and enhanced speed. Genes that were significantly different following the trajectory were found using the testPseudotime function of the TSCAN R package (v.1.36.0). In parallel, we also performed cell clustering within each CD8 subset using the Seurat function "FindNeighbors" (dims=1:20) and "FindCluster". To identify DEGs between the conditions HC and PD within each cluster of each subset, the function "FindAllMarkers" (log2FC.threshold = 0.5, test.use = "wilcox", min.pct = 0.3) was used. Selected DEGs of interest were visualized in heatmaps or violin plots. Of note, the pre-processing for cell clustering was slightly different from that of the pathway analyses aforementioned and the dedicated scripts were provided. The scripts used to analyze scRNA-seq data were available via https://doi.org/10.5281/zenodo.8395536; for single-cell clustering analysis, please check https://doi.org/10.5281/zenodo.8398047

For manuscripts utilizing custom algorithms or software that are central to the research but not yet described in published literature, software must be made available to editors and reviewers. We strongly encourage code deposition in a community repository (e.g. GitHub). See the Nature Portfolio guidelines for submitting code & software for further information.

Data

Policy information about availability of data

All manuscripts must include a data availability statement. This statement should provide the following information, where applicable:

- Accession codes, unique identifiers, or web links for publicly available datasets
- A description of any restrictions on data availability
- For clinical datasets or third party data, please ensure that the statement adheres to our policy

The raw scRNA-seq data generated in this study have been uploaded in NCBI GEO database under the accession number GSE237254 (https://www.ncbi.nlm.nih.gov/geo/query/acc.cgi?acc=GSE237254). The raw flow cytometry and mass cytometry (CyTOF) dataset generated in this work are available via a Zenodo repository (https://doi.org/10.5281/zenodo.8382970). The GRCh38-2020-A human reference genome used by CellRanger was downloaded from 10x Genomics website (https://support.10xgenomics.com/single-cell-gene-expression/software/release-notes/build#GRCh38_2020A). Source data are provided with this paper. Only the relevant clinical data (i.e., disease duration) that were displayed in figures are provided in Source Data. The disaggregated sex information is also available in Source Data.

Human research participants

Policy information about studies involving human research participants and Sex and Gender in Research.

Reporting on sex and gender	We have performed gender-specific analysis on different immune subsets, e.g., CD8 TEMRA, CD8 TCM (and the ratios between CD8 TEMRA and TCM), CD8 Treg (and the ratios between CD8 TEMRA and Treg), CD8 NKT, GZMA, Neutrophils, Eosinophils and ILC2. We have now shown them in Figure 1H, Figure 2C, 2J, Figure 3B, 3D, Figure 4H, 4J, 4L, 4M, 4N and Fig. S2L, S2M, S2Q, Fig. S3D. Of note, to reduce workload, we mainly re-examined those already showing highly-interesting results in the gender-mixed analyses. Unexpectedly, we indeed found a gender-biased observation on the indicated subsets or effector molecules. We have now updated the title and Abstract accordingly. We also selectively checked other effector molecules, such as serological GZMB, Perforin and others, but we did not notice significant results when analyzing female only samples (with no significance, we did not show them in this already-long paper). To avoid confusing the readers, we also did not show the gender-separated results of CD8 TEMRA for both CyTOF analysis and flow-cytometry analysis while only showing the corresponding flow-cytometry results. However, it is important to note that we have provided all the gender disaggregated values for each panel of each cytometry Figures in the accompanied Source Data table, which allows other investigators to check and re-analyze our work whenever needed.<br><br>It is also worth to highlight that for both CD8 TEMRA and CD8 TCM, we observed significant difference between PD and HC for both genders, although the results are much more striking in females (as reflected by the ROC analysis and significant values in gender-specific group comparisons). At the same time, one needs to consider the fact that males are much more susceptible to PD than females in general and we have analyzed 19 male while only 9 female PD patients in our cohort. When one interprets our results, one might need to keep this information in mind.<br><br>Sex was self-reported, supported by medical records and all the selected participants were examined by trained neurologists to remove any question in doubt. During the study design, we already considered the demographic fact that men are a higher risk for PD than women and therefore selected more men than women to participate this particular work.
Population characteristics	We recruited the participants from the ongoing nation-wide Luxembourg Parkinson's study with more than 800 PD and 800 HC (Hipp et al. 2018) (https://parkinson.lu/research-participation/luxembourg-parkinsons-study) and controlled for several major confounding factors, medications and comorbidities, known to affect the immune system, to ensure that our observations are PD-specific (for inclusion/exclusion details, refer to Table S1). Furthermore, we narrowed the patients to those with early-to-mid stage disease [Hoehn and Yahr (H&Y) staging scale: mean=2.3, ranging from 1.5 to 3.0; most of them were ≤2.5, except for five participants with a scale of 3]. Most of the selected patients had a disease duration of 10 years from clinical diagnosis while three of them had a disease duration of 12, 13 and 19 years, respectively. In the initial discovery cohort, we analyzed 28 PD patients (25 iPD aged 60-70 years and three genetic PD patients with mutations in GBA or PINK1) and 24 matched healthy controls (HC) (refer to "cohort design" in Materials and Methods, Table S1 and S2 for more details). Among 28 PD patients, 19 are males while 14 are males among 24 HC.<br><br>As a first validation, we analyzed cryopreserved available PBMC of another independent subcohort from the same Luxembourg Parkinson's study. 11 iPD and 12 age- and gender-matched HC were selected following the same inclusion/exclusion criteria as the discovery cohort. The average sampling age of iPD and HC is 64.90 and 63.46 years old, respectively. Eight out of 11 iPD were male while five out of 12 HC were male (for all the other information, please visit Table S2). Of note, due to the availability issue of sufficient suitable samples, we included two CMV seronegative participants who otherwise met with all the other selection criteria in the HC group of the validation cohort. Excluding these two samples from the

	analysis did not change our conclusion (see the peer review files). Also due to the sample availability issues, four HC participants were analyzed in both the initial discovery and validation analysis, but the cryopreserved samples from recent visits were used for the validation analysis, still showing certain independence even for these four samples. For scRNA-seq, considering our female-biased observation in CD8 T cells and comparability between groups, we selected only female participants (four HC and five iPD). The average sampling age of iPD and HC is 63.14 and 62.33 years old, respectively. All the iPD were selected for scRNA-seq with the H&Y staging scale of 2.0. For more details, please refer to Supplementary Table S2 and the text.
Recruitment	All study participants were recruited from the Luxembourg Parkinson's Study, a nation-wide, monocentric, observational longitudinal study with parallel healthy controls. The overall selection criteria are provided in Figure 1A and Supplementary Table 1. As a first step, we screened for HC and iPD patients aged 60-70 years (except the 3 genetic patients: one PD patient with two rare variants, one pathogenic homozygous variant N409S in GBA and another non-pathogenic heterozygous rare variant in PINK1 A383T, aged 48 years; one PD case with non-pathogenic heterozygous variant K13R in GBA, aged 55 years; one PD patient with the homozygous pathogenic variant L369P in PINK1, aged 45 years). Furthermore, we narrowed the patients to those with early-to-mid stage disease [Hoehn and Yahr (H&Y) staging scale: mean=2.3, ranging from 1.5 to 3.0; most of them ≤2.5, except for five participants with a scale of 3]. Since aging is the primary risk factor for PD and aging dramatically affects the immune system, we focused on a relatively narrow age window (60-70 years) in the PD and the corresponding HC group. We also excluded potential participants if they were diagnosed with any immune-associated diseases, such as diabetes, cancer, chronic inflammatory disease, autoimmune disease and acute infection or if they were currently treated with immunosuppressive medication (see Supplementary Table 1 for detailed overview of the exclusion criteria). After the first round of exclusion, 150 PD and 58 HC were further tested for their cytomegalovirus (CMV) serologic status based on biobanked serum samples. CMV has been well documented to facilitate the immunosenescence process. In order to make the immunological analysis comparable at such an advanced age, we only invited HC and PD subjects as participants for fresh blood analysis if they were seropositive for anti-CMV IgG. We invited only CMV seropositive participants for both technical and epidemiological reasons. Technically speaking, we could not exclude the possibility that some CMV negative participants might become CMV seropositive during the period between the previous serum sampling time and the current fresh blood sampling time. Second, according to a large-scale CMV seropositive investigation in the neighbor areas (Germany, most of the elderly are found CMV seropositive. Choosing CMV seronegative individuals at that age range would have further severely limited the availability of suitable samples. Therefore, we decided to first analyze the more-representative group of participants in their sixties. As a result, a total of 28 PD and 24 HC CMV positive individuals agreed to be included in this study requiring additional blood sampling (see Supplementary Table 2 for details on demographic and clinical information). To account for the circadian rhythm of immune cells trafficking throughout the body, all blood samples of the participants were collected in the morning between 8:00-11:00 AM and processed within six hours.<br><br>There is no obvious recruitment self-selection bias. Although both the clinical team and the experimental team work very closely and decided the recruitment inclusion/exclusion criteria together in this project, the clinical team is in charge of the patient selection and the clinical team even did not know what would be the potential clinical factors which might bias our immunological observations. But with our essential hypotheses that the immunological dysregulations might already occur in earlier stage of iPD, whenever we have to select patients, we chose those with lower H&Y scale values. We donot think this preference will affect our conclusion because this fits exactly with our main objective, i.e., to examine whether there is any peripheral immune dysregulatory events in early-to-mid stage patients.<br><br>For single-cell RNA-seq analysis, the patient selection is based on our female-biased observations in the initial discovery analysis and therefore only female patients were selected. Furthermore, considering the possibilities to only analyze a small number of samples in the single-cell RNA-seq analysis, we had to select a more homogeneous group. Therefore, we selected all the patients with a H&Y scale = 2. As the main objective of single-cell RNA-seq analysis is to study and characterize the underlying molecular mechanisms of our initial phenotype observations, our patient selection is also justified.
Ethics oversight	The study protocol (for the current project known as 'CoPImmunoPD') has been reviewed and approved by the Luxembourg National Research Ethics Committee (CNER) on 9th Oct 2019 (CNER approval Nr. 20140713-SU1; two additional amendment notifications received no objection decision on 20th Feb., 2023 and on 12th Apr., 2023, respectively). Informed consent was obtained before each participant was recruited into the study by the local clinical team in Luxembourg. There is no financial compensation for the participants.

Note that full information on the approval of the study protocol must also be provided in the manuscript.

Field-specific reporting

Please select the one below that is the best fit for your research. If you are not sure, read the appropriate sections before making your selection.

☒ Life sciences ☐ Behavioural & social sciences ☐ Ecological, evolutionary & environmental sciences

For a reference copy of the document with all sections, see nature.com/documents/nr-reporting-summary-flat.pdf

Life sciences study design

All studies must disclose on these points even when the disclosure is negative.

Sample size	The planned sample size in the study protocol was 30 PD vs 30 HC. Following all the various stringent inclusion and exclusion criteria (also considering the COVID-19 pandemic suddenly appeared in the middle of our recruitment), we were able to include 28 PD and 24 matched HC from >800 PD patients and >800 HC of our cohort in Luxembourg. In the initial discovery analysis, only fresh blood samples were analyzed.

During the revision, we performed a mini-validation with 11 iPD and 12 matched HC from cryopreserved PBMC.

We also selected 5 female iPD and 4 female HC for single-cell RNA-seq analysis also based on cyropreserved PBMC.

Data exclusions

Essentially, no data exclusion was done, except for the following cases caused by technical issues: in one flow cytometry staining panel, all Eomes related cell populations were excluded for two PD patients, because there was no ab staining for Eomes PE-Cy7; in another staining panel, two PD patients had no CD183 ab staining and therefore the related cell populations were removed for the further analysis. We also noticed that one PD patient visited twice during a short period. To avoid confusion, we decided to exclude the sample from one of the visits in the analysis. Although there is no obvious change on the overall results, we found the P-value showing negative correlation between disease duration following clinical diagnosis and CD8 TEMRA became slightly more significant (the p-value decreased from 0.054 to the current value of 0.0466). For CyTOF analysis, one PD was excluded to due to a wrong staining panel used ; one HC was excluded because of much fewer cells that were obtained due to technical mistakes during operation.

For soluble factor measurements, some of the cytokine values are missing because there was huge difference between the two technical duplicates, and the corresponding measurements were considered as non-reliable ones and were excluded.

In the validation cohort, for CD8 TCM frequency analysis, one PD was excluded as it was identified as an outlier by the default setting (Q=1%) of the recommended ROUT method of Graphpad Prism (v9).

Replication

Our initial findings were essentially based on fresh blood samples of one initial discovery subcohort of the Luxembourg Parkinson's study; We validated our key results in another subcohort of the Luxembourg Parkinson's study. Each participant (either patients or healthy controls) were biologically independent samples. Therefore, the number of participants in the given group were actually identical to the number of independent biological replicates. In another word, for the initial discovery analysis, we successfully repeated 28 and 24 times for patients and healthy controls, respectively. For the initial discovery analysis, as the participants visited us according to his or her schedule, we often need to repeated the measurements, separately for each participant using the same application setting, unless occasionally multiple selected participants visited our clinics in the same day by chance [when we analyzed multiple (up to four people due to the workload of staining five panels in parallel) samples together]. For the validation analysis, we successfully performed our analysis on 11 patients and 12 healthy controls. As we had to use cryopreserved PBMC, we were able to perform the staining and analysis only in two batches (split due to workload). As shown in the main or Supplementary Figures, all the results are reproducible even if different types of materials (frozen or fresh blood samples) were used. From this point of view, we have not only independent biological replications from different individual participants, but also validation at different technical layers.

We also performed single-cell RNA-seq analysis using 5 female iPD and 4 female HC based on cryopreserved PBMC in one batch. For single-cell RNA-seq analysis, our first attempt was failed technically, because of a substantial cell loss (>95%) after centrifugation and were even unable to proceed to sequencing steps (i.e., no RNA-seq data generation at all). But after solving the technical issues, the single-cell RNA-seq analysis is successful with all the analyzed samples.

Randomization

Randomization is impossible because we had to implement all the stringent inclusion and exclusion criteria to select PD or HC participants. Furthermore, we could not control and decide when the selected participants would visit the clinical team (for patients) or research nurses (for healthy controls).

Blinding

The experimental investigators were blind to the participant disease status before the analysis was finished. The investigators were not blind to the group allocation during data collection because an pseudoID without disclosing any personal identity information (e.g., PD123 or HC123; '123' is an exemplary ID) had to be first assigned to each participant by the research clinician. However, we do not think this will affect our results as the essential quantification equipment (cytometry) acquired immune cells unbiasedly (i.e., the investigators cannot influence the objective measurements/readouts on each of a large number of analyzed immune subsets for the given participant). Furthermore, for CyTOF analysis, the staining/analyzing investigators were even different from the mass spectrometry Helios operators who acquired all the cells and initially normalized the data. For cytokine multiplexing assay experiments and CMV IgG ELISA analysis, one also cannot influence the readouts of the corresponding quantification machine/platform. For single-cell RNA-seq analysis, blinding is impossible as we had to pool cells from different participants of the same group for each sorted CD8 subset. Furthermore, the people who performed sorting were different from those who prepared single-cell cDNA library, different from those who performed sequencing, and also different from those who performed computational analysis. Moreover, none of the operators can influence the objective readouts of their responsible steps (as a consequence, no one can influence the final results of the multiple-step single-cell RNA-seq analysis). In short, applying blinding analysis or not has no impact on our various machine-based objective measurements, analyses and conclusions in this work.

Reporting for specific materials, systems and methods

We require information from authors about some types of materials, experimental systems and methods used in many studies. Here, indicate whether each material, system or method listed is relevant to your study. If you are not sure if a list item applies to your research, read the appropriate section before selecting a response.

Materials & experimental systems

n/a	Involved in the study
[ ]	[x] Antibodies
[x]	[ ] Eukaryotic cell lines
[x]	[ ] Palaeontology and archaeology
[x]	[ ] Animals and other organisms
[ ]	[x] Clinical data
[x]	[ ] Dual use research of concern

Methods

n/a	Involved in the study
[x]	[ ] ChIP-seq
[ ]	[x] Flow cytometry
[x]	[ ] MRI-based neuroimaging

Antibodies

Antibodies used

All the used flow cytometry and mass cytometry antibodies (Abs) including the provider, cat number, specific metal isotype for mass cytometry or specific fluochromes for flow cytometry) were already included in Supplementary Table S3, S4 and S6. Of note, the Ab dilution factors used in the integrated standard CyTOF MDIPA kit was not available to the users by the manufacturer. All the Abs used for the cytotoxic marker cytometry analysis and CD8 subset sorting were already included in Supplementary Table S6. We provided them here again.

List 1 (refer to Supplementary Table S3). Mass Cytometry (CyTOF) antibodies used to analyze the whole blood immune compositions (the dilution factors of specific Abs used in standard integrated CyTOF MDIPA kit have already been pre-set by the manufacturer, which are unavailable to us).
Metal Isotope, Antibody, Clone, Manufacturer, Catalogue#
89Y, CD45, HI30, Fluidigm (now Standardbio), Part of MDIPA (cat. 201325)
103Rh, Live/Dead indicator, Fluidigm, Part of MDIPA, 201325
141Pr, CD196 (CCR6), G034E3, Fluidigm, Part of MDIPA, 201325
142Nd, CD117 (c-kit)*, 104D2, Biolegend, 313223
143Nd, CD123, 6H6 ,Fluidigm, Part of MDIPA, 201325
144Nd, CD19, HIB19, Fluidigm, Part of MDIPA, 201325
145Nd, CD4, RPA-T4, Fluidigm, Part of MDIPA, 201325
146Nd, CD8a, RPA-T8, Fluidigm, Part of MDIPA, 201325
147Sm, CD11c, Bu15, Fluidigm, Part of MDIPA, 201325
148Nd, CD16, 3G8, Fluidigm, Part of MDIPA, 201325
149Sm, CD45RO, UCHL1, Fluidigm, Part of MDIPA, 201325
150Nd, CD45RA, HI100, Fluidigm, Part of MDIPA, 201325
151Eu, CD161, HP-3G10, Fluidigm, Part of MDIPA, 201325
152Sm, CD194 (CCR4), L291H4, Fluidigm, Part of MDIPA, 201325
153Eu, CD25, BC96, Fluidigm, Part of MDIPA, 201325
154Sm, CD27, O323, Fluidigm, Part of MDIPA, 201325
155Gd, CD57, HCD57, Fluidigm, Part of MDIPA, 201325
156Gd, CD183 (CXCR3), G025H7, Fluidigm, Part of MDIPA, 201325
158Gd, CD185 (CXCR5), J252D4, Fluidigm, Part of MDIPA, 201325
159Tb, KLRG1*, SA231A2, Biolegend, 367702
160Gd, CD28, CD28.2, Fluidigm, Part of MDIPA, 201325
161Dy, CD38, HB-7, Fluidigm, Part of MDIPA, 201325
162Dy, CD336 (NKP44)*, P44-8, Biolegend, 325102
163Dy, CD56 (NCAM), NCAM16.2, Fluidigm, Part of MDIPA, 201325
164Dy, TCRgd, B1, Fluidigm, Part of MDIPA, 201325
165Ho, CD223 (LAG3), 11C3C65, Fluidigm, 3165037B
166Er, CD294, BM16, Fluidigm, Part of MDIPA, 201325
167Er, CD197 (CCR7), G043H7, Fluidigm, Part of MDIPA, 201325
168Er, CD14, 63D3, Fluidigm, Part of MDIPA, 201325
169Tm, CD49d*, 9F10, Biolegend, 304302
170Er, CD3, UCHT1, Fluidigm, Part of MDIPA, 201325
171Yb, CD20, 2H7, Fluidigm, Part of MDIPA, 201325
172Yb, CD66b, G10F5, Fluidigm, Part of MDIPA, 201325
173Yb, HLA-DR, LN3, Fluidigm, Part of MDIPA, 201325
174Yb, IgD, IA6-2, Fluidigm, Part of MDIPA, 201325
175Lu, CD279 (PD-1), EH12.2H7, Fluidigm, 3175008B, 201325
176Yb, CD127, A019D5, Fluidigm, Part of MDIPA, 201325

* in-house conjugation using Maxpar X8 Antibody Labeling Kits MDIPA (201325, Fluidigm). The predicted theoretical quantity used per reaction for the in-house conjugated Abs CD117-142Nd, KLRG1-159Tb, NKP44-162Dy, CD49d-169Tm, PD-1-175Lu and LAG3-165Ho was 0.5ug, 0.17ug, 0.5ug, 0.05ug, 0.17ug and 0.05ug, respectively. The theoretical concentration was calculated based on the expected average recovery rate of 60% for an antibody labelling procedure.

List 2 (refer to Supplementary Table S4). Flow cytometry antibodies used to stain the PBMCs of participants analyzed in this study.

Ab Target, Fluorochrome, Dilution factor, Manufacturer, Reference, Clone
Fc Blocking Abs , 1:50, BD, 564765, Fc1 (no azide and low endotoxin)
CD3*, BUV737, 1:100, BD, 741822, HIT3a
CD3*, BV510, 1:100, BD, 564713, HIT3a
CD4, BUV395, 1:100, BD, 563550, SK3
CD8, BUV496, 1:100, BD, 564804, RPA-T8
CD25, BV786, 1:50, BD, 741035, 2A3
CD25, BB515, 1:50, BD, 564467, 2A3
CD27, BB700, 1:50, BD, 566450, M-T271
CD28, BUV785, 1:50, BioLegend, 302950, CD28.2
CD31, BV605, 1:50, BD, 562855, WM59
CD39, BV711, 1:50, BioLegend, 328228, A1
CD45RA, BV421, 1:50, BioLegend, 304130, HI100
CD45RA, BV785, 1:50, BioLegend, 304140, HI100
CD45RO, PE-CF594, 1:50, BD, 562299, UCHL1
CD57, FITC ,1:50, BD, 555619, NK-1
CD71, FITC ,1:50, BioLegend, 334104, CY1G4
CD98, BV786, 1:50, BD, 744507, UM7F8
CD122, PE, 1:50, BioLegend, 339006 ,TU27
CD127 (IL7R), BV421, 1:50, BD, 562436, HIL-7R-M21
CD127 (IL7R), BV711, 1:50, BioLegend, 351328, A019D5
CD183 (CXCR3), PE, 1:50, BD, 560928, 1C6/CXCR3
CD194 (CCR4), APC, 1:50, BioLegend, 359408, L291H4
CD196 (CCR6), PE-Cy7, 1:50, BD, 560620 ,11A9
CD197 (CCR7), BV421, 1:50, BioLegend, 353208, G043H7
CD223 (LAG3), BV711, 1:50, BioLegend, 369320, 11C3C65
CD278 (ICOS), BV605, 1:50, BioLegend, 313538, C398.4A
CD279 (PD-1), BV605, 1:50, BioLegend, 329924 ,EH12.2H7
GLUT1, PE ,1:500, Abcam, ab209449, EPR3915
KLRG1, PE-Cy7, 1:50, BioLegend, 368614, 14C2A07
Intracellular markers:
CD152 (CTLA4), PE-Cy5, 1:20, BD, 555854, BNI3
FOXP3, APC ,1:20, BioLegend, 320114, 206D
Phospho S6, AF488, 1:20, CST, 4803S, D57.2.2E
Helios, Pacific Blue ,1:20, BioLegend, 137220, 22F6
Ki-67, Alex488 ,1:20, BD, 561165, B56
GATA3, PE-Cy7 ,1:20, BD, 560405, L50-823
RORγT, BV650, 1:20, BD, 563424, Q21-559
T-bet, PE, 1:20, BioLegend, 644810, 4B10
Eomes, PE-Cy7, 1:20, Thermo Fisher Scientific, 25-4877-42, WD1928
Live/Dead, APC-Cy7, 1:500, Thermo Fisher Scientific, L34976 /
*, different fluorochromes in the list above might be used in different staining panels as we employed five staining panels in parallel.

List 3 (Refer to Supplementary Table S6): Antibodies used for the cytotoxic marker analysis via FCM and for CD8 subset sorting via FACS.
Marker, Fluorochrome, Dilution factors, Manufacture, Reference, Clone, Application, Extracellular or intracellular, Lot number
CD8, BUV496, 1/100, BD, 612942, RPA-T8, Cytotoxic & Sorting Extracellular, 2213972
CD4, BUV395, 1/100, BD, 563550, SK3, Cytotoxic & Sorting Extracellular,1313997
CD45RA, BV785, 1/50, Biolegend, 304140, HI100, Cytotoxic &Sorting Extracellular, B369489
CD3, BV510, 1/20, BD, 564713, HIT3a, Cytotoxic & Sorting Extracellular,2129056
CCR7, BV421, 1/50, Biolegend, 353208, G043H7, Cytotoxic & Sorting Extracellular,B361376
CD45RO, PE-CF594, 1/50, BD, 562299, UCHL1, Cytotoxic& Sorting Extracellular, 1333677
Perforin, FITC, 1/30 ,Biolegend, 353310, B-D48, Cytotoxic Intracellular, B336273
Granzyme B (GZMB), RY586, 1/750, BD, 568133, GB11 Cytotoxic Intracellular, 2056267-1
Granzyme A (GZMA), Alexa Fluor 700, 1/150, Biolegend, 507210, CB9 Cytotoxic Intracellular, B322484
Granzyme K (GZMK), Alexa Fluor 647, 1/30, BD, 566655, G3H69 Cytotoxic Intracellular, 2234680
Of note, for the extracellular mix, the final volume was 50 µL diluted in Brilliant stain buffer. For the intracellular mix, the final volume was 100 µL diluted in permeabilization buffer.

Validation

Reaction species and applications of all the primary Abs used in this study have been validated by the given manufactures, as directly stated in the specific datasheet of the given abs. Furthermore, a all the Abs were purchased from leading reliable manufactures (BD, Biolegend, CST or Thermo Fisher Scientific, Abcam or Fluidigm/Standardbio), the general routine validation statements for flow cytometry or mass cytometry analysis are also available on the website of the corresponding provider. For BD, please visit: https://www.bdbiosciences.com/en-lu/products/reagents/flow-cytometry-reagents/research-reagents/quality-and-reproducibility; For Biolegend, please visit: https://www.biolegend.com/en-us/quality/quality-control; For Abcam, please visit: https://www.abcam.com/primary-antibodies/how-we-validate-our-antibodies#Flow%20cytometry; For CST, please visit: https://www.cellsignal.com/about-us/

our-approach-process/antibody-validation-flow-cytometry.

For Fluidigm/StandardBio, as only the MDIPA kit was used, please directly visit the specific validation statement here: https://www.standardbio.com/asset/259.

As we only used one primary abs from Thermo Fisher Scientific, please directly visit: https://www.thermofisher.com/antibody/product/EOMES-Antibody-clone-WD1928-Monoclonal/25-4877-42.

Clinical data

Policy information about clinical studies

All manuscripts should comply with the ICMJEguidelines for publication of clinical research and a completedCONSORT checklist must be included with all submissions.

Clinical trial registration	The overall activity related to our Luxembourg Parkinson's Study cohort has been registered under NCT05266872 at ClinicalTrials.gov (https://clinicaltrials.gov/ct2/show/study/NCT05266872). But for this specific immunology work, we did not register again for this particular observational work.
Study protocol	The full study protocol including updated amendments is provided with the manuscript submission.
Data collection	All the PD patients were recruited at the Parkinson Research Clinic of the Centre Hospitalier de Luxembourg (CHL) and all the healthy controls (HC) were recruited at the Clinical and Epidemiological Investigation Centre (CIEC) of Luxembourg Institute of Health but still under the coordination of the Luxembourg Parkinson's study. The first sampling for the participants invited for fresh blood analysis occurred on Feb 2020 while the last one took place on May 2021. For the independent validation subcohort, we directly analyzed the available cyropreserved PBMC following the ethical approvals of secondary usages and sample access approvals.
Outcomes	The major expected outcomes are:<br>o Identification of idiopathic PD-related changes in immune cell subsets, which was assessed by CyTOF (mass cytometry) and flow cytometry, followed by statistical tests between PD and HC groups for each of the quantified immune cells.<br>o Identification of idiopathic PD-related changes in serological cytokines or chemokines, which was assessed by MSD cytokine multiplexing assays, followed by statistical tests between PD and HC groups for each of the quantified cytokines/chemokines.<br>o Identification of significant association between immune cell subsets with the selected PD disease stages, which was assessed by calculating potential Spearman correlation between various immune cell subsets quantified by our cytometry analysis and available quantitative clinical datasets from each individual patient.<br>o Identification of significantly-enriched key genes or pathways or subnetworks changed in the particular disturbed immune subsets with the selected PD disease stages, which was assessed by single-cell RNA-seq analysis with differential expression gene analysis between PD and HC and pathway enrichment analysis, clustering analysis within different CD8 T-cell subsets.<br>o Identification of immune changes associated with the selected known-PD genetic mutations, e.g., particular LRRK2 mutations, which was assessed by statistical analysis of various immune subsets between those genetic PD and other PD patients (although we didnot observe a clear specific pattern related to those genetic PD patients).<br>o Identification of subgroups of idiopathic PD-related immunological changes, which was assessed by PCA analysis and gender-specific analysis for all the analyzed immune features.

Flow Cytometry

Plots

Confirm that:

- [x] The axis labels state the marker and fluorochrome used (e.g. CD4-FITC).
- [x] The axis scales are clearly visible. Include numbers along axes only for bottom left plot of group (a 'group' is an analysis of identical markers).
- [x] All plots are contour plots with outliers or pseudocolor plots.
- [x] A numerical value for number of cells or percentage (with statistics) is provided.

Methodology

Sample preparation	For each study participant, 1 million of fresh PBMCs were stained for each of the 5 staining panels. Prior to the cell staining, the PBMCs were incubated for 15 min at 4°C with 50 µL Brilliant Stain Buffer (BD, 563794), containing 2.5 µL Fc blocking antibodies (BD, 564765). 50 µL of 2 x concentrated surface antibody master mixes diluted in Brilliant Stain Buffer were added to the cell suspension and incubated for 30 min at 4°C (Table S4). Following three washing steps with FCM buffer (300 x g, 5 min, 4 °C), the stained PBMCs were fixed for 60 min at RT using the fixation reagent of the True-Nuclear Transcription Factor Buffer Set (Biolegend, 424401). After fixation, the cells were centrifuged (400 x g, 5 min, 4°C), re-suspended in 200 µL FCM buffer and left at 4°C overnight. The next day, the PBMCs were washed once in permeabilization buffer of the same kit and re-suspended in permeabilization buffer, containing 2.5 µL Fc blocking antibodies. After a 10-min incubation, the cells were centrifuged and the cell pellet was re-suspended in 100 µL permeabilization buffer containing the antibodies for the intracellular targets for a 30-40 min incubation at RT. Finally, the cells were washed three times in permeabilization buffer and re-suspended in 100 µL of FCM buffer for the acquisition on a BD LSRFortessa.<br><br>For flow-cytometry-based cytotoxic capacity analysis, biobank-cryopreserved PBMC were thawed in a water bath at 37°C

until the last ice crystal was visible, and they were then rapidly transferred into a 15 mL Falcon tube containing 4 mL of heat-inactivated FBS (Gibco, 10500-064) at room temperature (RT). After centrifugation (500 x g, 5 min, RT), PBMC were washed at RT with 10 mL of FCM buffer (PBS 1X, 2%FBS; Fisher Scientific, PBS without Ca/Mg/Phenol red, 20012-027). After a second centrifugation step, the cells were counted in 1 mL of FCM buffer. One million cells per participant were re-suspended in 100 µL of PBS 1X and transferred into a 96-well U-bottom plate (Greiner, M9436-100Ea). Following two washing steps with 1X PBS, PBMC were incubated at RT with 100 µL of Zombie NIR Live/dead staining reagent (Biolegend, 423106, dilution 1/200 in 1X PBS) for 30 min. 100 µL of FCM buffer were added and the plate was centrifuged (500 x g, 4°C, 5 min) and washed a second time. Prior to the cell staining, PBMC were incubated for 15 min at 4°C with 50 µL of Brilliant Stain Buffer (BD, 563794), containing 2.5 µL of Human Fc Block (BD, 564765). 50 µL of surface antibody master mix diluted in Brilliant Stain Buffer were added to the cell suspension and incubated for 30 min at 4°C (Table S6). Following two washing steps with cold FCM buffer (500 x g, 5 min, 4 °C), the stained PBMC were fixed for 60 min at 4°C using 200 µL of True-Nuclear Transcription Factor Buffer Set (Biolegend, 424401). PBMC were washed twice with the permeabilization buffer of the same kit and re-suspended in 50 µL of permeabilization buffer containing 2.5 µL of Human FC Block. After a 10-min incubation at 4°C, the cell pellet was re-suspended in 100 µL of permeabilization buffer containing the antibodies for the intracellular targets and was incubated for 30 min at 4°C (Table S6). Finally, the cells were washed twice in cold permeabilization buffer and re-suspended in 100 µL of a freshly-prepared 4% Formaldehyde solution (Fisher Scientific, 10751395) for 30 min at 4°C. After centrifugation, the cell pellet was re-suspended with 200 µL of cold FCM buffer. All the stained PBMC were acquired on a BD FACSymphony A6 (BD Biosciences) and the FCS files were analyzed using the FlowJo v10.8.0 software. The detailed gating strategy is illustrated in Figure 5.

Fluorescence activated cell sorting (FACS) was performed using a BD FACSymphony S6 six-way cell sorter with a 100 µm nozzle. After cell counting, PBMC were stained with the protocol used to characterize the cytotoxic phenotype, but without proceeding to the intracellular staining steps. To preserve cell integrity to a maximum degree, the 15-mL Falcon collection tubes were extemporaneously pre-coated with a cold solution of 20 % BSA in 1X PBS for at least one hour. Before sorting, the pre-coating solution was discarded and replaced with 500 µL of 0.04% FBS in PBS at 4°C until sorting. The sorting experiment was performed on the samples from four HC and five PD to separate four subsets of CD8 T cells. The gating strategy (also refer to Supplementary Fig. 6A, Figure 6A) was performed as follows. The lymphocyte population was determined by their characteristic of forward and side scatter properties and then the doublets were excluded. After the identification of live cells and CD3 T-cells, the CD8+CD4- cells were selected. Among these cells, four subsets were identified as follows: CD8 Naïve (CD45RO-CCR7+), CD8 TCM (CD45RO+CCR7+), CD8 TEM (CD45RO+CCR7-) and CD8 TEMRA (CD45RO-CCR7-). Samples from the same group (HC, n=4 or iPD, n=5) for each subset were pooled and centrifuged at 4°C 400g for 10 min. Discard supernatant, keep cells in 100ul 0.5% BSA/PBS at 4°C and proceed to single cell isolation and sequencing.

Instrument

BD LSRFortessa for fresh blood analysis. For intracellular cytotoxic analysis, we used BD FACSymphony A6. For FACS sorting, we used BD FACSymphony A6 six-way sorter. For CyTOF analysis, Helios was used.

Software

The flow cytometry acquisition software: BD FACSDiva 8.0.1;
For CyTOF acquisition software: CyTOF Helios software v7.0.8493;
The sorting acquisition software: FACSDiva v9.5;
The analysis software: FlowJO V10

Cell population abundance

The purity check was carried out per subset, immediately post sorting per patient with a mean value of 82.93%, 82.90%, 92.29% and 84.91%, respectively for CD8 naive, CD8 TCM, TEM and CD8 TEMRA (for marker definition, please refer to the text and the sample preparation section above). To further increase the reliability of our analysis, for scRNA-seq, we removed all the individual cells from the further analysis, showing the CCR7 mRNA expression levels mismatched with the expected patterns of the corresponding CCR7+ or CCR7- subsets (for details, please refer to the Methods of the manuscript).

Gating strategy

We have already provided various gating strategy examples in Figure 5, Figure 6 and Supplementary Figure 1, 5 and 6. For the flow cytometry analysis, we first gated on total lymphocytes, then singlets, then living cells, then total CD3 T cells, then CD4 or CD8 T cells, then all the various combinations using various markers (as listed in the Supplementary Figure 6A and Figure 6A). For the mass cytometry (CyTOF) analysis, we gated on all non-beads living singlets for further analysis. The detailed gating strategy of CyTOF analysis was provided in Supplementary Figure 1.

For sorting, the gating strategy (also refer to Supplementary Fig. 6A, Figure 6A) was performed as follows. The lymphocyte population was determined by their characteristic of forward and side scatter properties and then the doublets were excluded. After the identification of live cells and CD3 T-cells, the CD8+CD4- cells were selected. Among these cells, four subsets were identified as follows: CD8 Tn (CD45RO-CCR7+), CD8 TCM (CD45RO+CCR7+), CD8 TEM (CD45RO+CCR7-) and CD8 TEMRA (CD45RO-CCR7-).

☒ Tick this box to confirm that a figure exemplifying the gating strategy is provided in the Supplementary Information.
